# Epidemiological Aspects and World Distribution of HTLV-1 Infection

**DOI:** 10.3389/fmicb.2012.00388

**Published:** 2012-11-15

**Authors:** Antoine Gessain, Olivier Cassar

**Affiliations:** ^1^Département de Virologie, Unité d’épidémiologie et physiopathologie des virus oncogènes, Institut PasteurParis, France; ^2^CNRS, URA3015Paris, France

**Keywords:** HTLV-1, HTLV-1 epidemiology, HTLV-1 world distribution, HTLV-1 in Europe, HTLV-1 in Africa, HTLV-1 in the Americas, HTLV-1 in Asia, HTLV-1 in Oceania

## Abstract

The human T-cell leukemia virus type 1 (HTLV-1), identified as the first human oncogenic retrovirus 30 years ago, is not an ubiquitous virus. HTLV-1 is present throughout the world, with clusters of high endemicity located often nearby areas where the virus is nearly absent. The main HTLV-1 highly endemic regions are the Southwestern part of Japan, sub-Saharan Africa and South America, the Caribbean area, and foci in Middle East and Australo-Melanesia. The origin of this puzzling geographical or rather ethnic repartition is probably linked to a founder effect in some groups with the persistence of a high viral transmission rate. Despite different socio-economic and cultural environments, the HTLV-1 prevalence increases gradually with age, especially among women in all highly endemic areas. The three modes of HTLV-1 transmission are mother to child, sexual transmission, and transmission with contaminated blood products. Twenty years ago, de Thé and Bomford estimated the total number of HTLV-1 carriers to be 10–20 millions people. At that time, large regions had not been investigated, few population-based studies were available and the assays used for HTLV-1 serology were not enough specific. Despite the fact that there is still a lot of data lacking in large areas of the world and that most of the HTLV-1 studies concern only blood donors, pregnant women, or different selected patients or high-risk groups, we shall try based on the most recent data, to revisit the world distribution and the estimates of the number of HTLV-1 infected persons. Our best estimates range from 5–10 millions HTLV-1 infected individuals. However, these results were based on only approximately 1.5 billion of individuals originating from known HTLV-1 endemic areas with reliable available epidemiological data. Correct estimates in other highly populated regions, such as China, India, the Maghreb, and East Africa, is currently not possible, thus, the current number of HTLV-1 carriers is very probably much higher.

## Introduction

Very rapidly after HTLV-1 discovery and its association with adult T-cell leukemia (ATL), several studies were initiated both by American and Japanese researchers, to get insights into the distribution, the transmission modes, as well as the origin of HTLV-1.

Thus, as soon as 1982–1984, important works demonstrated clearly that Japan was a high endemic area for HTLV-1. Interestingly, since the first studies, the geographic distribution of HTLV-1 carriers is quite uneven in Japan and the greatest prevalence is observed in Southwestern Japan (Kyushu island and the Okinawa archipelago). The origin of such a peculiar distribution is still the matter of several hypotheses (Ishida and Hinuma, 1986; Miura et al., 1994; Eguchi et al., 2009; Watanabe, 2011).

Nearly concomitantly to the initial papers by the Japanese groups, the US teams demonstrated that the Caribbean area was also endemic for HTLV-1. Soon after, ATL patients were reported in the Caribbean community living in the United Kingdom (Catovsky et al., 1982). Such data, together with some early epidemiological studies showing an HTLV-1 seroprevalence in serum samples from inhabitants originating from different African countries, indicated then that such a retrovirus was also endemic in some areas of the African continent (Biggar et al., 1984; Hunsmann et al., 1984; Saxinger et al., 1984; de-The et al., 1985; de The and Gessain, 1986; Delaporte et al., 1989a,b, 1991; Ouattara et al., 1989; Verdier et al., 1989, 1994; Goubau et al., 1990; Dumas et al., 1991; Schrijvers et al., 1991).

The simian counterpart of HTLV-1 was also rapidly discovered, based on the findings of STLV-1 in several monkey species from Asia and Africa (Hunsmann et al., 1983; Guo et al., 1984; Hayami et al., 1984; Homma et al., 1984; Becker et al., 1985; Watanabe et al., 1985; Blakeslee et al., 1987; Koralnik et al., 1994).

The different transmission modes of HTLV-1 have been rapidly studied by the same groups (Kinoshita et al., 1984; Okochi et al., 1984; Hino et al., 1985; Kajiyama et al., 1986; Murphy et al., 1989; Takahashi et al., 1991; Hino, 2011). They are now rather well understood. This led to some efficient preventive measures in several countries (Ando et al., 1987; Hino et al., 1987; Inaba et al., 1989).

In contrast, the world distribution and the global and loco-regional estimation of the HTLV-1 prevalence remain yet poorly known. This lack of knowledge is mainly due to four different factors: (1) Several large regions/areas have not been investigated for HTLV-1 infection. Thus, the prevalence remains barely unknown in many areas of the world. This is evident in some highly populated regions of Asia or in East and North Africa. (2) The assays used for HTLV-1 serology exhibited some lack of specificity leading in the 1980s–1990s to an overestimated HTLV-1 prevalence. (3) Most of the works performed to appreciate the HTLV-1 prevalence are based on study of series of blood donors, pregnant women, or hospitalized patients. Population-based studies trying to estimate HTLV-1 prevalence in large areas, or even at a country level, remain very rare. (4) A very important last point is that, in most of the studied areas, HTLV-1 distribution is not homogeneous. HTLV-1 is indeed present mainly as relatively small foci or clusters with a high or very high prevalence of infection, with nearby quite low endemic areas. This has been very well exemplified in Southern Japan and in some areas of South America. Thus, to give a precise estimation of HTLV-1 prevalence in a specific country or area is relatively difficult and, in some cases, nearly impossible.

Very few studies have thus given an estimation of the HTLV-1 world prevalence. Japan and the African continent have been rapidly considered, as the two regions in which HTLV-1 infected persons were the most numerous. Then, South America has also been considered as a very important focus of HTLV-1 carriers and associated diseases (mainly ATL or tropical spastic paraparesis/HTLV-1 associated myelopathy (TSP/HAM), a chronic severe neuromyelopathy). One of the most frequently quoted estimation number of infected persons is based on a paper, published in 1993 by G. de Thé and R. Bomford from our group in Pasteur (de The and Bomford, 1993). This study gave a rough estimation of 10–20 millions of HTLV-1 infected individuals worldwide. In most of the review published since then, this number is often taken as a fact (Proietti et al., 2005; Verdonck et al., 2007; Hlela et al., 2009; Watanabe, 2011).

By writing this paper, our main goal was to revisit the situation of HTLV-1 epidemiology, especially its world distribution, and its estimated prevalence. This will be based on the current data, available in 2012, thus around 30 years after the first published epidemiological studies and nearly 20 years after the last estimation of global HTLV-1 prevalence.

This ambitious work has been based on the analyses of: (1) most of the 1,100 papers referenced in PubMed on such a topic, (2) most of the available book chapters concerning HTLV-1 epidemiology but also (3) on all the abstracts (of the epidemiology sections) of the International Conference on HTLV and related viruses held since 1985.

This study was, of course, possible thanks to our expertise and experience in the field since 30 years for one of us and nearly 10 years for the other one.

After presenting the different methods used for the epidemiological studies of HTLV-1 infection, we shall describe briefly the major epidemiological determinants and the different transmission modes of this human retroviral infection. Then, a small chapter will report the major findings concerning the molecular epidemiology of HTLV-1, which possesses an unusual great genetic stability for a retrovirus. Lastly, we shall try to present, with a table and a map, the HTLV-1 worldwide distribution and its prevalence estimates by continent and by country when possible.

## Methods for HTLV-1 Epidemiology

Most of the studies were performed on series of blood donors, pregnant women, or hospitalized patients. In few cases, population-based studies were done in some villages, towns, or even regions of a given country. The epidemiological and demographic characteristics of blood donors could be very different according to the countries: indeed, they can be quite representative of the middle-class population in some countries but in other areas, either they are mainly family members of hospitalized patients, or they originate from low socio-economic populations and are sometimes paid to give blood and, lastly, in several areas (especially in some African studies) they are mainly young men. HTLV-1 prevalence varies according to age, sex, and economic level in most of HTLV-1 endemic areas. Thus, the prevalence found in blood donors can be, of course, useful, but provides not always the best data to estimate precisely the HTLV-1 prevalence in a given country. The real prevalence is very probably higher than that found in blood donors in most of the cases. Thus, the data based on pregnant women are generally more useful to compare the situation between different areas or countries as first, it is quite representative of a given region and second, the mean age of pregnant women is generally comparable (about 22–26 years) in most of the countries. The studies in general populations, in or out-adult patients, can also be very useful to try to appreciate the HTLV-1 prevalence in a given area as, in the great majority, the tested patients do not suffer of the very rare diseases linked specifically to HTLV-1 infection.

Diagnostic methods used for the study of HTLV-1 infection include mainly serological assays searching for antibodies directed specifically against different HTLV-1 antigens. Screening tests are usually Enzyme-Linked Immunoabsorbent Assay (ELISA) or Particle Agglutination (PA). Confirmatory tests are immunofluorescence (IFA), but mostly Western Blot (WB), or Innogenetics line immunoassay (INNO-LIA). Moreover, research of integrated provirus, in the DNA from peripheral blood cells, could be done by qualitative and/or quantitative polymerase chain reaction (PCR). Despite some improvements in the WB assays specificity during the last two decades, indeterminate serological patterns remain frequent following WB analysis, and represent an important concern for routine screening in blood banks in Europe, the Americas, and some parts of Africa. It is also of course a major issue for comparative analyses between epidemiological studies performed in both low and high endemic areas, especially in intertropical areas (Mauclere et al., 1997; Filippone et al., 2012). The significance of these frequent indeterminate WB can be various but, in the majority of the cases, remains mostly unknown and a matter of debate reviewed in Filippone et al. (2012). Indeed, in rare cases, these patterns have been associated to (i) HTLV-1 but mostly HTLV-2 infection exhibiting an atypical HTLV-serology, (ii) HTLV-1 seroconversion, (iii) infection by a different retrovirus such as the recently discovered HTLV-3 or HTLV-4 (Mahieux and Gessain, 2009). Furthermore, some have been considered as the results of cross-reactivity against other microbial agents, especially *Plasmodium falciparum* in Central Africa and Indonesia (Porter et al., 1995; Mahieux et al., 2000). In this review, we shall take into account, in the great majority, only the studies in which HTLV-1 infection has been confirmed by a specific test, mostly a WB.

## Major Epidemiological Determinants of HTLV-1

HTLV-1 is not an ubiquitous virus. Indeed, it is present throughout the world, with clusters of high endemicity located often nearby areas where the virus is nearly absent. In these foci, the HTLV-1 seroprevalence in adults is estimated to be at least 1–2% but it can also reach 20–40% in persons older that 50 years in some specific clusters. The main very highly endemic areas are the Southwestern part of Japan, some parts of the Caribbean area, and its surroundings regions, foci in South America including especially parts of Colombia and French Guyana, some areas of intertropical Africa (such as South Gabon) and of the middle East (such as the Mashad region in Iran), and rare isolated clusters in Australo-Melanesia. In Europe, only Romania seems to represent an HTLV-1 endemic region. The origin of this puzzling geographical or rather ethnic repartition is not well understood but is probably linked to a founder effect in some groups, followed by the persistence of a high viral transmission rate.

Interestingly and despite different socio-economic and cultural environments, HTLV-1 seroprevalence increases gradually with age, especially in women, in all the highly endemic areas. The general increase with age may be related to a cohort effect, as well as demonstrated in Japan, while the increase in women might also be due to an accumulation of sexual exposures with age (Blattner et al., 1986; Chavance et al., 1989; Ueda et al., 1989; Mueller, 1991; Murphy et al., 1991).

Three modes of transmission have been demonstrated for HTLV-1. (1) Mother to child transmission, which is mainly linked to a prolonged breast-feeding after 6 months of age (Hino, 2011). Ten to 25% of the breast-fed children born from HTLV-1 infected mothers will become infected. High level of HTLV-1 proviral load in milk, in blood cells as well as high HTLV-1 antibody titers in the serum, and long duration of breast-feeding (at least >6 months) represent major risk factors for HTLV-1 transmission from mother to child (Takahashi et al., 1991; Hino et al., 1994; Ureta-Vidal et al., 1999; Li et al., 2004). (2) Sexual transmission, which mainly, but not exclusively, occurs from male to female, and is thought to be responsible for the increased seroprevalence with age in women (Murphy et al., 1989; Stuver et al., 1993; Takezaki et al., 1995; Kaplan et al., 1996; Roucoux et al., 2005). (3) Transmission with contaminated blood products (containing HTLV-1 infected lymphocytes), which is responsible for an acquired HTLV-1 infection among a high proportion (15–60%) of the blood recipients (Okochi et al., 1984; Inaba et al., 1989). HTLV-1 infection is also present among intravenous drug users but at a lesser extent than HTLV-2 (Murphy et al., 1999).

## HTLV-1 Molecular Epidemiology: Presence of Geographical/Ethnic Related Subtypes/Genotypes

On a molecular point of view, HTLV-1 possesses a remarkable genetic stability, an unusual feature for a retrovirus. Viral amplification via clonal expansion of infected cells, rather than by reverse transcription is, very probably, the reason for this striking genetic stability. The low sequence variation of HTLV-1 can be used as a molecular tool to follow the migrations of infected populations in the recent or distant past and thus to gain new insights into the origin, evolution, and modes of dissemination of such retroviruses and of their hosts (Gessain et al., 1992b; Yanagihara, 1994). The few nucleotide substitutions observed among virus strains are indeed specific to the geographic origin of the patients rather than the pathology. Four major geographic subtypes (genotypes) have been reported. They include the Cosmopolitan subtype A, the Central African subtype B, the Central African/Pygmies subtype D, and the Australo-Melanesian subtype C. A limited number of strains found in Central Africa belong to other rare subtypes (E, F, G). The Cosmopolitan subtype A, which comprised several geographical subgroups (Japanese, West Africa, North African…) is the most widespread, being endemic in Japan, the Caribbean area, Central and South America, North and West Africa as well as part of the Middle East. The sequence variability within subtype A is very low. This is very likely to reflect a relatively recent dissemination (some centuries to few millenium) of this genotype from a common ancestor. The Australo-Melanesian subtype C is the most divergent. This result reflects a long period of evolution (at least several millenium) in isolated populations living in different islands of the Pacific area. The appearance of these HTLV-1 subtypes in humans was strongly suggested to be linked to interspecies transmission between STLV-1 infected monkeys and humans, followed by variable period of evolution in the human host (Salemi et al., 1998; Vandamme et al., 1998; Meertens et al., 2001; Nerrienet et al., 2001; Wolfe et al., 2005). Indeed, STLV-1, the simian counterpart of HTLV-1, infects several species of non-human primates (NHPs) of the Old Word, ranging from chimpanzees, orangutans, and gorillas to mandrills, as well as several African small monkey species and a wide range of macaques, and other Asian monkeys. Interestingly, STLV-1 infection was also associated to the development of ATL in some NHPs.

**Figure 1:**
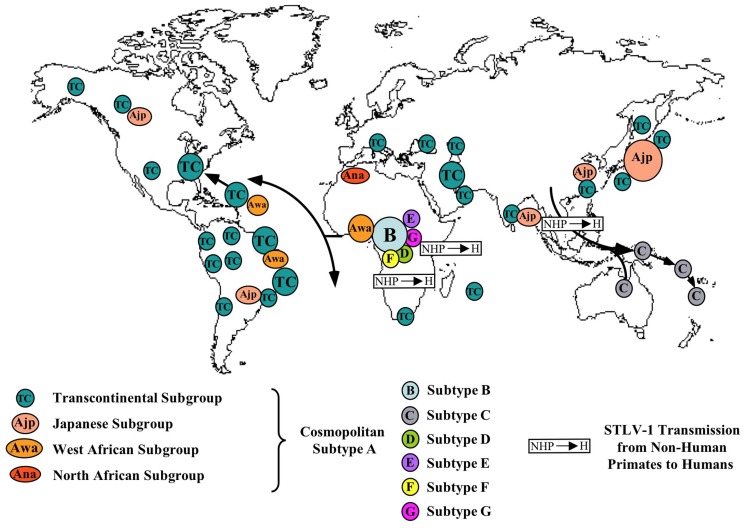
**Map of the geographical distribution of HTLV-1 subtypes (A–G), and the main modes of viral dissemination by movements of infected populations**. Small arrows indicate the very probable interspecies transmission of STLV-1 (S) from monkeys to Humans (H) at the origin of some current HTLV-1 subtypes. These different subtypes comprise the Cosmopolitan A subtype with its different subgroups: TC (Transcontinental being the most frequent and widespread one), Awa (West African), Ana (North African), Ajp (Japanese), B or Central African being the most frequent in this large endemic area, C or Australo-Melanesian D, also from Central Africa and present especially in certain Pygmy groups and lastly E, F, G with very few strains yet reported (all in Central Africa). The main HTLV-1 molecular epidemiological studies used to draw this map are the following ones: (Gessain et al., 1991; Gasmi et al., 1994; Miura et al., 1994; Mahieux et al., 1997, 1998; Salemi et al., 1998; Vandamme et al., 1998; Wolfe et al., 2005; Cassar et al., 2007; Gessain, 2011).

A map of the different HTLV-1 molecular subtypes and places of possible interspecies transmissions from STLV-1 infected monkeys to humans is presented on Figure 1.

There is so far no solid evidence that either a particular specific mutation or a genotype is associated with the development of a TSP/HAM or an ATL, in an asymptomatic carrier. Data concerning level of transmission or proviral load according to molecular subtypes need to be performed.

## HTLV-1 Worldwide Distribution and Prevalence Estimates by Geographical Area

**Figure 2:**
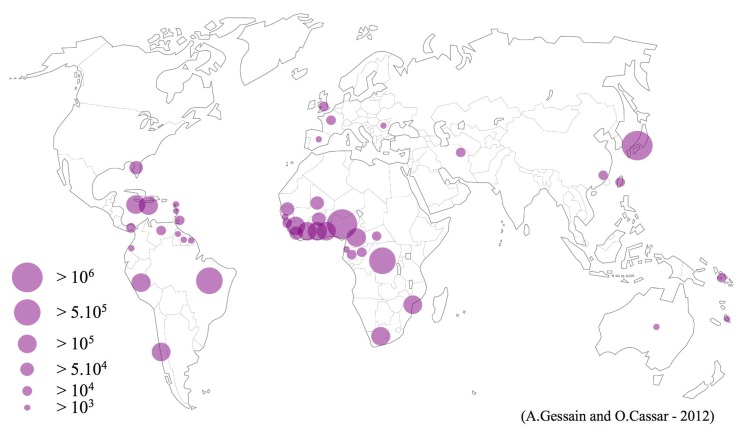
**Geographical distribution of the main foci of HTLV-1 infection**. Estimates of the number of HTLV-1 infected carriers, based on approximately 1.5 billion of individuals from known endemic areas and reliable epidemiological data obtained from studies among pregnant women and/or blood donors and/or different adult populations. In few countries, HTLV-1 endemic areas are limited to residents of certain regions such as Mashad in Iran, The Fujian Province in China, Tumaco in Colombia and Central Australia.

To give a general overview of the HTLV-1 worldwide distribution was relatively easy for most of the regions. However, to try to provide an estimate, even rough, of the number of HTLV-1 infected persons in the different world areas, even in well-studied high HTLV-1 endemic regions was not at all an easy task. In all the cases, such estimates must be absolutely taken with cautious. Indeed, as said above, very few large population-based studies have been done and most of the analyzed studies concern mainly blood donors, pregnant women, or populations of different in or out-adult patients not really representative of the population of a given area or country on an epidemiological point of view (Table 1 and Figure 2).

**Table 1:** **HTLV-1 seroprevalence among pregnant women originating from HTLV-1 endemic countries in Africa, West Indies, North and South America, Asia, and Europe**.

Region	Country	HTLV-1 seropositive women (%)	Reference
AFRICA	Guinea-Bissau	2.2 (27/1,231)	Andersson et al. (1997)
	Côte d’Ivoire	1.9 (10/513)	Verdier et al. (1989)
	Ghana	2.1 (20/960)	Armah et al. (2006)
	Burkina-Faso	1 (5/492)	Collenberg et al. (2006)
	Nigeria	5.5 (20/364)	Olaleye et al. (1995)
	Gabon	2.1 (19/907)	Etenna et al. (2008)
	Zaire	4.6 (19/414)	Goubau et al. (1993)
	Zaire	3.7 (43/1,166)	Delaporte et al. (1995)
	Congo	0.7 (14/2,070)	Tuppin et al. (1996)
	South Africa	0.2 (1/428)	Goubau et al. (1993)
WEST INDIES	Martinique	2.4 (17/716)	Denis et al. (1988)
	Martinique	1.9 (9/467)	Mansuy et al. (1999)
	Jamaica	3.5 (81/2,329)	Wiktor et al. (1993)
	Jamaica	3.8 (350/9,226)	Maloney et al. (2006)
	Haiti	2.2 (11/500)	Allain et al. (1992)
	Haiti	4.2 (12/287)	Tortevoye et al. (2005)
NORTH AMERICA	Mexico (Yucatan)	0 (0/590)	Gongora-Biachi et al. (1996)
ASIA	India (Vellore)	0 (0/201)	Ramalingam et al. (2001)
	Japan (Honshu)	0.6 (14/2,414)	Goto et al. (1997)
	Japan (Nagasaki and Kyushu)	3.7 (187/5,015)	Hino et al. (1985)
	Japan (whole Kyushu)	5.4 (885/16,283)	Oki et al. (1992)
	Japan (Kagoshima in Kyushu)	5.8 (138/2,374)	Umemoto et al. (1994)
	Japan (Okinawa)	3.9 (670/17,207)	Maehama (2004)
EUROPE	United Kingdom	0.04 (52/126,010)*	Ades et al. (2000)
	United Kingdom	0.03 (23/69,013)°	Ades et al. (2000)
	France	0.1 (12/10,398)	Courtois et al. (1990)
	Italy	0.02 (1/6,000)*	Taylor et al. (2005)
	Spain	0.01 (2/20,366)*	Machuca et al. (2000)
	Slovenia	0.01 (1/10,369)	Poljak et al. (1998)
	Greece	0 (0/2,016)	Tseliou et al. (2006)
SOUTH AMERICA	French Guyana (Noir Marron population)	5.7 (70/1,225)	Carles et al. (2004)
	French Guyana (Noir Marron population)	4.2 (181/4,266)	Tortevoye et al. (2005)
	Brazil (Mato Grosso)	0.2 (7/2,965)	Ydy et al. (2009)
	Brazil (Mato Grosso South)	0.1 (37/32,512)	Figueiro-Filho et al. (2007)
	Brazil (Salvador Bahia)	0.8 (57/6,754)	Bittencourt et al. (2001)
	Brazil (Sao Paulo)	0.1 (1/913)	Olbrich Neto and Meira (2004)
	Brazil (Sao Luis)	0.2 (4/2,044)	Guimaraes de Souza et al. (2012)
	Peru (Andes,Coast,Jungle,Lima)	1.3 (32/2,492)	Alarcon et al. (2006)
	Peru (Quechua population)	2.4 (5/211)	Zurita et al. (1997)
	Argentina (Cordoba-Central)	0.1 (3/3,143)	Trenchi et al. (2007)

*The quoted studies are, to our knowledge, among the most representative and reliable ones to date*.

*including black African and Afro-Caribbean women.

*°Women born exclusively in the United Kingdom*.

### HTLV-1 in Europe

Europe represents around 400 millions of persons. Numerous epidemiological studies have been performed in this continent, mainly in blood donors and in pregnant women (Courtois et al., 1990; Courouce et al., 1993; Nightingale et al., 1993; Zaaijer et al., 1994; Dalekos et al., 1995; Ferrante et al., 1997; Hale et al., 1997; Tuset et al., 1997; Poljak et al., 1998; Ades et al., 2000; Machuca et al., 2000; Tseliou et al., 2004, 2006; Vrielink and Reesink, 2004; Taylor et al., 2005; Davidson et al., 2006; Laperche et al., 2009; Brant et al., 2011). Furthermore, several large series of ATL and TSP/HAM have been reported (Gout et al., 1989; Rio et al., 1990; Plumelle et al., 1993; Martin et al., 2010; Ceesay et al., 2012). The most studied countries are the United Kingdom (UK), Metropolitan France, and Spain. Based on all these works, it is now clear that most (at least more than 80%) of the persons infected by HTLV-1 living currently in Europe originate directly, or are children or descendents of immigrants, from a high endemic area, mostly West Indies and Africa. Thus, in UK, most of HTLV-1 carriers originate from the former British West Indies, especially Jamaica and also, at a lesser extent, Barbados, Trinidad, and Tobago (Catovsky et al., 1982; Payne et al., 2004; Dougan et al., 2005). More rarely, they originate from West Africa as Ghana or Sierra Leone. Concerning Metropolitan France, most of the persons originate from French West Indies as Martinique or Guadeloupe (Gout et al., 1989; Gessain et al., 1990c; Rio et al., 1990; Plumelle et al., 1993). They can also originate from West or Central Africa as Senegal, Mali, Guinea, Côte d’Ivoire, or Cameroon (Gessain et al., 1990b, 1992a; Rio et al., 1990; Mahe et al., 1994; Duval et al., 2010). It is important to note that in blood donors and, thus, very probably in the general population, a certain proportion of infected persons are women, of Caucasian background, that have acquired HTLV-1 through sexual contacts with a partner originating from an HTLV-1 endemic area. This situation can represent around 5–10% in different European countries including France or UK (Dougan et al., 2005). In Spain, the situation is slightly different as, due to historical reasons, most of the HTLV-1 infected persons are from South America (Toro et al., 2002; Padua et al., 2011; Trevino et al., 2012). However the prevalence seems much lower than in UK or in Metropolitan France. In Portugal, this is about the same but, in some cases, the HTLV-1 infected persons can originate from ancient African colonies such as Mozambique and Angola (Padua et al., 2011). Intravenous drug users represent also a population relatively endemic for HTLV-1, specially in Spain, Italy, and Ireland even if HTLV-2 is always much more prevalent in such a specific population (Gradilone et al., 1986; Calderon et al., 1995; Chironna et al., 1996).

The level of HTLV-1 infection in most of the other European countries is very low as demonstrated by an HTLV-1 prevalence in first-time blood donors of less than 0.4/10,000 and a great rarity of ATL or TSP/HAM reports (Laperche et al., 2009). The only exception seems to be Romania, which appears to be the only true HTLV-1 endemic area in Europe (Paun et al., 1994; Veelken et al., 1996). Indeed, a prevalence of 5.3/10,000 donations has been recently reported among first-time blood donors in this East European country (population of 22 millions; Laperche et al., 2009). HTLV-1 infection in polytransfused patients is also quite high ranging from 3 to 25%. Furthermore, series of cases of ATL or TSP/HAM have been reported in Romania. Lastly, sporadic cases have also been seen in immigrants from this country living in other European countries (Veelken et al., 1996; Manca et al., 1997). These data, taken together, demonstrate clearly a relatively high level of HTLV-1 infection in this country. The origin of such situation remains unknown and the matter of different hypotheses.

The estimation of the number of HTLV-1 infected persons in Europe is a very difficult exercise. Based on all data from UK and especially their numerous studies, the team of Graham Taylor suggests that 20,000–30,000 people have HTLV-1 infection in the UK (Ades et al., 2000). Concerning Metropolitan France, based on the study on prevalence in blood donors, pregnant women, and the incidence of ATL cases, we can estimate that it is roughly equivalent with around 10,000–25,000 HTLV-1 infected persons. Importantly and, as in the UK, most of them are undiagnosed. Concerning Romania, this is more difficult, due to the few available studies. However, in a recent study, the prevalence in the first-time blood donors seems 10 times more in Romania than in UK and Metropolitan France (Laperche et al., 2009). A rough minimal estimation of few thousands with a wide range (3,000–15,000) can be proposed. However, we cannot rule out the presence of possible HTLV-1 clusters, or foci, in some specific areas or populations in such country.

### HTLV-1 in Africa

The total population of Africa is estimated at a little more than 1 billion in 2012. The African population has grown a lot over the past century. Indeed, the population doubled for the period 1982–2009 and quadrupled during the years 1955–2009. Thus, more than 40% of the population is below 15 years old in most sub-Saharan countries. Africa is probably the largest endemic area for HTLV-1. However, despite numerous epidemiological studies and reports of sporadic cases or even small series of ATL and TSP/HAM cases, the situation concerning the level of HTLV-1 infection, is not really known in several countries and regions of this large continent. Indeed, very few large studies on representative general population are available.

Here, we shall first briefly report the situation, based on the most solid studies (large populations of mainly blood donors, pregnant women, or adult hospitalized patients or control groups, and robust serological tests including mainly a WB for confirmation), according to five large geo-climatological areas: North Africa, West Africa, Central Africa, East Africa, and lastly South Africa. Then, we shall try to estimate the number of HTLV-1 infected persons in some specific countries for which reliable data are available.

#### North Africa/Maghreb

The very few studies concern mainly blood donors or multi-transfused patients from Egypt, Morocco, and Tunisia where the HTLV-1 seroprevalence appears to be very low or negative (Saxinger et al., 1984; de-The et al., 1985; de The and Gessain, 1986; El-ghazzawi et al., 1987; el-Farrash et al., 1988; van Doornum et al., 1990; Mojaat et al., 1999; Kawashti et al., 2005; Stienlauf et al., 2009). Very few cases of ATL or TSP/HAM have been reported in such countries, including at least Morocco and Egypt, either diagnosed locally or in immigrants, especially in France (Thyss et al., 1990; Gasmi et al., 1994; Gharibi et al., 2011). In summary, based on the scarce available data, Morocco seems to be, in North Africa, the only country that can be considered as HTLV-1 endemic, however at a low level and a reliable estimation of the number of HTLV-1 infected persons in North Africa is currently impossible.

#### West Africa

This large area comprises 16 countries: Benin, Burkina-Faso, Cape Verde, Côte d’Ivoire, Gambia, Ghana, Guinea and Guinea-Bissau, Liberia, Mali, Mauritania, Niger, Nigeria, Senegal, Sierra Leone, and Togo. This represents more than 300 millions of inhabitants. Several large studies have been performed in Senegal, Guinea-Bissau, Côte d’Ivoire, Ghana, and Nigeria. Furthermore, ATL and/or TSP/HAM have been reported in most of these countries including especially Senegal, Mauritania, Mali, Côte d’Ivoire, Nigeria (Fleming et al., 1986; Gessain et al., 1990a,c; Williams et al., 1993, 1994; Mahe et al., 1994; Michel et al., 1996; Analo et al., 1998; Fouchard et al., 1998; Sene-Diouf et al., 2000). The HTLV-1 seroprevalence in blood donors ranged mainly from 0.2 to 3%. However, in most of these studies, blood donors are mainly young men. In Senegal, based on a study of 4,900 blood donors, HTLV-1 seroprevalence was of 0.16%, however most of the infected donors originated from the South of the country, where the prevalence reached 1% (Diop et al., 2006). In Guinea, it was of 1.2% (22/1,785; Gessain et al., 1993a), while in Côte d’Ivoire and Ghana it ranges from 0.5 to 2% depending on the studies (Ouattara et al., 1989; Verdier et al., 1989; Biggar et al., 1993; Sarkodie et al., 2001; Ampofo et al., 2002; Armah et al., 2006). In Nigeria, it varies from 0.7 to 3.7% according to geographical location (Fleming et al., 1986; Analo et al., 1998). Other studies were performed on pregnant women or representative general adult population or on different control groups. The HTLV-1 prevalence was around 1% in Guinea, with variations from 0.2 to 1.9% depending on the region (Jeannel et al., 1995), and ranged from 2.2 to 3.6% in Guinea-Bissau (Andersson et al., 1997; Larsen et al., 2000; da Silva et al., 2009). It was of 1.2% in Gambia (Del Mistro et al., 1994) and ranged from 0.2 to 1.2% in Senegal (Hunsmann et al., 1984). It was of 1% in Burkina-Faso (Collenberg et al., 2006) and ranged from 1 to 1.6% in Liberia, Togo and Benin (Hunsmann et al., 1984; Dumas et al., 1991; Verdier et al., 1994; Houinato et al., 1996), and from 1 to 2.7% in Côte d’Ivoire and Ghana according to the type of studies (Ouattara et al., 1989; Verdier et al., 1989, 1994; Biggar et al., 1993; Goubau et al., 1993; Armah et al., 2006) and reached 5.5% in Nigeria (Olaleye et al., 1994, 1995). The estimates of the number of HTLV-1 infected persons in most of the West African countries are given in Table 2.

**Table 2:** **Estimates of the number of HTLV-1 infected carriers, based on approximately 1.5 billion of individuals from known HTLV-1 endemic areas and reliable epidemiological data obtained from studies performed among pregnant women and/or blood donors and/or different adult populations**.

Continent/Country	Population!	HTLV-1 range	
**AFRICA**			
Senegal	12,969,606	30,000	105,000
Gambia	1,840,454	2,500	13,000
Guinea-Bissau	1,628,603	12,000	28,000
Guinea	10,884,958	75,000	150,000
Sierra Leone/ Liberia	5,485,998/3,887,886	50,000	100,000
Côte d’Ivoire	21,952,093	130,000	250,000
Ghana	25,241,998	125,000	375,000
Togo/Benin	6,961,049/9,598,787	80,000	160,000
Burkina Fasso	17,275,115	42,000	125,000
Mali	14,533,511	32,000	95,000
Nigeria	170,123,740	850,000	1,700,000
Cameroon	20,129,878	80,000	180,000
Eq. Guinea	685,991	1,500	4,500
Gabon	1,608,321	16,000	30,000
CAR	5,057,208	15,000	30,000
DRC	73,599,190	600,000	1,300,000
Republic of The Congo	4,366,266	12,000	36,000
Mozambique	23,515,934	120,000	360,000
South Africa	48,810,427	180,000	540,000
**ASIA**			
China (Fujian Province)	35,110,000	2,000	20,000
Japan*	127,368,088	1,080,000	1,300,000
Iran (Mashad area)	78,868,711	10,000	40,000
Taiwan	23,113,901	10,000	30,000
**EUROPE**			
United Kingdom**	63,047,162	20,000	30,000
France	65,630,692	15,000	25,000
Spain	47,042,984	1,000	8,000
Romania	21,848,504	3,000	15,000
**CARIBBEAN REGION**			
Haiti/Dominican Republic	9,801,664/10,088,598	150,000	350,000
Jamaica	2,889,187	100,000	140,000
Guadeloupe^#^	401,730	3,000	6,000
Martinique^#^	395,953	3,000	6,000
Trindad and Tobago	1,226,383	9,000	18,000
**NORTH AMERICA**			
United States	313,847,465	90,000	100,000
**CENTRAL AND SOUTH AMERICA**			
Panama	3,510,045	3,500	25,000
Colombia (Tumaco area)	45,594 (est.1988)	1,000	1,500
Venezuela	28,047,938	14,000	70,000
Suriname	560,157	3,000	7,000
French Guyana	217,000	2,000	5,000
Guyana	741,908	2,000	5,000
Peru	29,549,517	150,000	450,000
Brazil	205,716,890	300,000	800,000
Chile	17,067,369	90,000	250,000
**AUSTRALO-MELANESIA**			
Australia (Aboriginal Australians)	463,900	2,500	5,000
Solomon Islands	584,578	3,000	6,000
Vanuatu	227,574	250	1,000
Total	1,567,570,505	4,520,250	9,295,000

*The HTLV-1 ranges were estimated combining the prevalence rates deduced from the above studied populations and the global repartition of individuals by age and sex in each studied country. In some highly populated countries and regions, such as China, India, the Maghreb and East Africa, correct estimates are currently not possible. Thus, the current total number of HTLV-1 carriers in the world is very probably much higher. Eq. Guinea, Equatorial Guinea; CAR, Central African Republic*.

*!According to the CIA World Factbook 2012 estimations (www.cia.gov)*.

*According to Satake et al. (2012).

**According to Brant et al. (2011).

*^#^According to the National Institute of Statistics and Economic Studies 2011 estimations (www.insee.fr)*.

#### Central Africa

This area comprises nine countries including, Angola, Cameroon, Central African Republic (CAR), Chad, Democratic Republic of the Congo (DRC) formerly Republic of Zaire, Equatorial Guinea, Gabon, Republic of the Congo, Sao-Tome-et-Principe. This represents around 130 millions of persons. Several important epidemiological studies have been performed in Cameroon and especially in Gabon. Cases of ATL and/or TSP/HAM have been reported mainly in Cameroon, Gabon and the DRC (Kayembe et al., 1990; Jeannel et al., 1993). HTLV-1 prevalence in adults living in Cameroon ranges globally from 0.5 to 2% depending on the geographical location, the studied population, and the ethnic group (Mauclere et al., 1997; Mauclère et al., 2011; Filippone et al., 2012). Indeed, the Pygmies groups, who live mainly in the Southern region of this country, have a much higher level of HTLV-1 prevalence (around of 2–10% in adults depending on age) than the Bantu groups (around 0.3–1.5%), who represent most of the population of this area (Mauclere et al., 1997; Mauclère et al., 2011; Filippone et al., 2012). In Gabon, several studies of different representative urban or rural populations have been performed (Delaporte et al., 1988, 1991; Bertherat et al., 1998). This is the best-studied country for Africa concerning the HTLV-1 infection. All the data indicated a global high HTLV-1 seroprevalence of around 5–10% in the adult populations (Delaporte et al., 1988, 1991; Bertherat et al., 1998). In pregnant women it ranged from 1 to 5% according to the geographical location (Schrijvers et al., 1991; Etenna et al., 2008). Based on several studies, it appears clearly that the Haut-Ogoué region (a Southeastern area of the country) is the highest endemic area of Gabon for HTLV-1 infection. Indeed, in some villages of this region, the HTLV-1 seroprevalence can reach more than 25% in elder adults, especially women (Delaporte et al., 1989b; Le Hesran et al., 1994). This area represents very probably the highest yet known HTLV-1 endemic area in the African continent. In CAR, HTLV-1 infection has been reported in Pygmies from the Southern region (Gessain et al., 1993b) and a seroprevalence of 7% has been recently shown in aged adults (>55 years old) of the same rain forest area (Pepin et al., 2010). In the Republic of the Congo and in DRC, HTLV-1 prevalence in pregnant women ranged from 0.7 to 3.7% depending on the studies (Delaporte et al., 1995; Tuppin et al., 1996). Furthermore, several other reports from DRC indicated that a relatively high HTLV-1 prevalence (5 to more than 20%) in different adult populations including hospitalized patients, prostitutes, leprosy patients (Kayembe et al., 1990; Wiktor et al., 1990; Delaporte et al., 1995; Lechat et al., 1997). Such results depended widely on the geographical areas of the tested persons with high prevalence region (as the Haut-Zaire/Equateur) and low prevalence ones (Goubau et al., 1993; Delaporte et al., 1995). The prevalence of HTLV-1 in Chad seems quite lower (around 0.5–1% in adults in the Southern part). The situation in Angola is less known. The estimates of the number of HTLV-1 infected persons in Cameroon, Gabon, Equatorial Guinea, CAR, DRC and the Republic of The Congo are given in Table 2.

#### East Africa

This large area comprises 12 countries including: Burundi, Erythrea, Ethiopia, Kenya, Malawi, Mozambique, Rwanda, Somalia, Tanzania, Uganda, Zambia, and Zimbabwe. Relatively few studies on HTLV-1 infection (with the exception of Mozambique) have been performed in such a large and highly populated region (total of more than 330 millions of inhabitants). Moreover, only sporadic cases of ATL and/or TSP/HAM have been reported from East African countries. Lastly, this region appears globally much less endemic for HTLV-1 than West and Central Africa. The reasons of such a situation are unknown. Indeed, all the studies performed in the countries, North of Malawi, indicate a very low or an absence of HTLV-1 infection. As examples, a nationwide community-based survey performed in Rwanda on more than 2,500 samples indicated an HTLV-1 seroprevalence of 0.2% (Group, 1989), and in Zimbabwe, only one blood donor out of 931 (0.1%) was found HTLV-1 seropositive (Houston et al., 1994). The situation in Mozambique (a country of 24 millions) appears different. Indeed, a study on 2,019 blood donors from Maputo city reported an HTLV-1 seroprevalence of 0.9% (Gudo et al., 2009). Another study in blood donors found an HTLV-1 seroprevalence rate, among 15- to 49-year-old men and women respectively of 0.9 and 1.2% (Cunha et al., 2007). A study on 752 individuals attending public heath centers from different areas of this country found a seroprevalence of 2.3% with geographical regional variation (Caterino-de-Araujo et al., 2010). Furthermore, some cases of TSP/HAM have been reported in Mozambique (Engelbrecht et al., 1999) and HTLV-1 infection has been seen at a rate of around 4% in HIV infected patients (Bhatt et al., 2009). The estimation of the number of HTLV-1 infected persons can be done only for Mozambique (Table 2).

#### South African region

This area comprises only three countries: Botswana, Namibia, and South Africa. Several studies have been performed but concern only South Africa, a country of 48 millions of inhabitants.

Indeed, several sporadic case or series of ATL, TSP/HAM, or infective dermatitis have been reported in South African patients (Bhigjee et al., 1990, 1993; Joubert et al., 1991; Jogessar et al., 1992). Indeed, as soon as 1999, around 200 black patients with a TSP/HAM have been seen in Durban. Furthermore, several studies performed in blood donors indicated an HTLV-1 seroprevalence ranging from 0 to 5% according to the type of tested population (black, white, young men, geographical location…; Bhigjee et al., 1993). A community-based seroprevalence survey done in adults from Natal/KwaZulu found a seroprevalence of 2.6% (26/1,018; Bhigjee et al., 1993). Similar results (0.5–3%) have been found in adults in other districts of this state and other states of the country (Bhigjee et al., 1994; Taylor et al., 1996; van der Ryst et al., 1996). The estimate of the number of HTLV-1 carriers in South Africa is given on Table 2.

Concerning the islands of the Indian Ocean, very few data are available. The situation is barely unknown in Madagascar, Comoros, and Mauritius but, to our knowledge, no case of ATL or TSP/HAM has been reported from these countries. It is different for the Reunion Island (an overseas French department of approximately 800,000 inhabitants) and the Seychelles archipelago. Indeed, in the Reunion Island, despite that few cases of TSP/HAM and ATL have been reported (Cnudde et al., 1991; Mahieux et al., 1994), a study on 3,900 blood donors (mostly young adult males) indicated only one infected person (Mahieux et al., 1994). By contrast, in the Seychelles, a cluster of TSP/HAM cases has been described as well as an HTLV-1 seroprevalence of 3–5% in blood donors (Roman et al., 1987; Lavanchy et al., 1991).

### HTLV-1 in the Americas

#### North America

Most of the available studies on HTLV-1 prevalence in the United States, concern blood donors and intravenous drug users. In one of the first large study, nearly 40,000 blood donors in eight geographically diverse areas were screened for HTLV-1. Only 10 of them (0.025%) showed evidence of HTLV-1 infection (Williams et al., 1988). Seroprevalence rates ranged from 0 to 0.1% at the locations sampled, with HTLV-1 antibodies found predominantly in donors from the Southeastern and Southwestern United States. The Retrovirus Epidemiology Donor Study group (REDS) evaluated, during 1991–1995, the HTLV-1 prevalence among 1.7 million donors from five REDS blood centers to 0.009% (Schreiber et al., 1997). In 2001, another study reported that the HTLV-1 seroprevalence rate among 21,000 individuals representing various patient populations including blood donors was 0.02% (Poiesz et al., 2001). HTLV-1, but mostly HTLV-2, infections are highly endemic among intravenous drug users in certain urban areas of the country (e.g., New Jersey), with seroprevalence rates reaching nearly 20% (Freeman et al., 1995). Among them, African-Americans were significantly more likely than Hispanics and other racial groups to be HTLV-1 positive (Lee et al., 1990; Cantor et al., 1991). The first patient with ATL, initially thought to have a Sezary’s syndrome and from whom HTLV-1 was first isolated, was described in 1980 in the United States (USl Poiesz et al., 1980). Typical ATL cases were detected among US native-born patients (Blayney et al., 1983; Dosik et al., 1988) and the first description of ATL in members of a single family from the US occurred in 1988 (Denic et al., 1988). Concerning TSP/HAM, small series of cases were published since 1988 (Bhagavati et al., 1988; Janssen et al., 1991). Later, larger series of ATL and TSP/HAM cases have been described in the US and most of the cases were identified in African-American individuals from the Southeastern states such as Florida (Harrington et al., 1995). A study estimates the number of HTLV-1 and 2 infected persons at around 260,000 and that they are likely more than 3,600 people in the United States with unrecognized TSP/HAM (Orland et al., 2003). It remains difficult to provide a solid estimate for HTLV-1 in the USA (Table 2).

HTLV-1 seems to be rare in Canada and the number of HTLV-1 cases is unknown. Nevertheless, the HTLV-1 prevalence observed among 168,668 blood donors in the Toronto region was 0.02 and 2.3% among regional individuals and people of Caribbean origin respectively (Chiavetta et al., 1992). HTLV-1 infection was also described in Amerindians from the coastal regions of British Columbia (Dekaban et al., 1994; Picard et al., 1995; Peters et al., 2000). Furthermore, some associated diseases (ATL and TSP/HAM) were reported in coastal British Colombia Indians (Power et al., 1989; Oger et al., 1993; Dekaban et al., 1994; Gascoyne et al., 1996).

In Mexico, there is little information on HTLV-1 prevalence in the general population despite the large population size of this country which counts nearly 115 millions inhabitants. The few studies are mainly conducted in the Yucatan peninsula or some different states (Nuevo Leon in Northeast Mexico) and show the absence of HTLV-1 infection among healthy women as well as in blood donors (Gongora-Biachi et al., 1996, 1997; Trejo-Avila et al., 1996).

#### Central America

Central America consists of seven states (Belize, Costa Rica, El Salvador, Guatemala, Honduras, Nicaragua, and Panama). In 2012, its estimated population is 42.6 millions. Despite the fact that some of these countries have strong commercial and cultural ties with the highly HTLV-1 endemic Caribbean Islands, relatively few studies concerning the HTLV-1 infection and the associated diseases have been performed except in the Honduras, Panama and, to a lesser extent, in Costa Rica. In these countries, the HTLV-1 seroprevalence is rather low but with significant differences between the populations tested. Thus, the HTLV-1 prevalence is significantly higher in non-Mestizo population, especially individuals living in the coastal cities of Honduras, than in the Mestizo ethnic groups (8.1 versus 0.5% respectively; de Rivera et al., 1995). Additional studies showed an overall HTLV-1 prevalence of 0.3% in the general Honduran population who live in central and Northwestern part of the country (Segurado et al., 1997) and a higher HTLV-1 prevalence in black natives individuals, without known risk factors, but originating from cities located on the Atlantic coast (Vallejo et al., 1996). In Panama, HTLV-1 seroprevalence varied from 0.2 to 2% in the general adult population throughout the country (Reeves et al., 1990). Few ATL or TSP/HAM cases have been reported from Honduran native-born individuals (Temple et al., 1986) and in the Panamanian population (Levine et al., 1989; Gracia et al., 1990).

The situation regarding HTLV-1 in Nicaragua and Costa Rica is poorly known but rare studies revealed similar relatively low HTLV-1 prevalence rates (0.2–0.7%), among the Nicaraguan and Costa Rican adult populations (Khabbaz et al., 1990; Qiu et al., 2008). In Belize, Guatemala and El Salvador, no HTLV-1 record about the prevalence of the HTLV-1 infection has been published to our knowledge.

#### South America

This area comprises 13 countries including Argentina, Bolivia, Brazil, Chile, Colombia, Ecuador, French Guyana (an overseas region of France), Guyana, Paraguay, Peru, Surinam, Uruguay, and Venezuela. This represents around 400 millions of persons. Numerous studies on HTLV-1 prevalence have been performed in many of these countries, especially in Brazil, Peru, Columbia, Chile, Argentina, and French Guyana. This vast continent represents, as a whole, a major endemic area for HTLV-1 infection and associated diseases. Even more than in other world regions, it is very difficult to appreciate the general HTLV-1 prevalence in a given country, as specific high endemic foci have been frequently reported in several South American countries. This is exemplified in Peru, in some of the Quechua population groups, and also in Columbia (Tumaco area) or in French Guyana (Noir-Marron population), where some specific groups of African origin are highly endemic for HTLV-I infection and associated diseases.

In Peru, a real multiethnic country mainly inhabited by Mestizos and Amerindians, HTLV-1 prevalence in blood donors ranges from 1.2 to 1.7% depending on the region (Quispe et al., 2009) and from 1.3 to 3.8% in pregnant women or in the adult general population (Zurita et al., 1997; Sanchez-Palacios et al., 2003; Alarcon et al., 2006). Furthermore several large series of TSP/HAM and ATL have been reported (Gotuzzo et al., 2004; Beltran et al., 2011). However, as noted above, several studies have clearly demonstrated that persons of Amerindians origin (Quechua speaking groups), often living in isolated areas, are highly HTLV-1 endemic (Fujiyoshi et al., 1999; Einsiedel et al., 2010).

In Chile, mainly populated by persons of Spanish ancestry mixed with various Amerindian groups, the situation appears, by certain aspects, roughly similar than in Peru. Indeed, HTLV-1 seems more endemic among indigenous people from isolated Amerindians groups, living in the Andes, or in the most Southern region of the country, than in the general population. Studies indicated an HTLV-1 seroprevalence ranging from 0.7 to 1.9% in blood donors (Vasquez et al., 1991). Large series of TSP/HAM (more than 200 cases seen before 1991) have been reported in Chilean patients (Cartier et al., 1989, 1992) while ATL were more rarely described (Cabrera et al., 1991).

In Columbia, several studies were focused on Tumaco, a densely populated island of the Pacific lowland recognized as having a very high prevalence of TSP/HAM (Arango et al., 1988; Zaninovic et al., 1988). In the general population of this specific focus (mostly inhabited by persons of African ancestry), the overall prevalence rate of HTLV-1 was 2.8% and reached 5.3% in the adults (Trujillo et al., 1992). Other reports indicated the presence of some foci of HTLV-1 infection in different isolated Indian populations (Zaninovic et al., 1994). Several ATL cases have also been described in patients from Columbia (Blank et al., 1993).

In Venezuela, based on the few available data, the HTLV-1 seroprevalence seems quite low. Indeed, it was of 0.2% in a large series of blood donors from Caracas (Leon et al., 2003). Furthermore, only rare cases of TSP/HAM have been reported (Zabaleta et al., 1994).

In Paraguay, HTLV-1 is rare and seems absent among the Paraguayan general healthy population (Zoulek et al., 1992). In Urugay, the seroprevalence reached 0.75% among the Urugayan blood donors (Muchinik et al., 1992).

French Guiana has been extensively studied for HTLV-1 infection (Kazanji and Gessain, 2003). In this French overseas region, HTLV-1 is mainly found in the Noir-Marron population, an ethnic group of African ancestry, also present currently in Surinam (Tortevoye et al., 2000, 2005). Indeed, this population is highly endemic for HTLV-1 (Plancoulaine et al., 1998; Carles et al., 2004) and several ATL cases have been reported in this ethnic group (Gerard et al., 1995). HTLV-1 infection and few associated diseases have also been reported in Guyana, and Surinam mainly in the persons of African ancestry (Pouliquen et al., 2004).

The numerous studies performed on blood donors and pregnant women from different areas of Argentina have revealed globally a low or very HTLV-1 seroprevalence, ranging roughly from 0.01 to 0.2% depending on the type and geographical location of the tested populations (Gastaldello et al., 2004; Trenchi et al., 2007; Berini et al., 2010). Some regions are thus considered as non-endemic (as the central areas) in contrast to the Northeastern provinces of the country (Biglione et al., 2005). In the same line, relatively few cases of TSP/HAM and ATL have been reported in this country (Biglione et al., 2003) ATL cases seems to be only described in individuals of Caucasian origin (Gioseffi et al., 1995; Marin et al., 2002).

Concerning Brazil, the situation is quite different and based on a large amount of solid publications, we can consider without any doubt that this country of more than 200 millions of inhabitants represents one of the largest endemic area for HTLV-1 and associated diseases. Indeed, several hundreds of cases of TSP/HAM, ATL, as well as large series of infective dermatitis cases have been reported in Brazilian patients (de Oliveira et al., 1990; Pombo De Oliveira, 1996; Araujo et al., 1998; Bittencourt et al., 2009). Furthermore, several studies performed on large populations of blood donors have found an heterogeneous HTLV-1 seroprevalence ranging from 0.04 to 1% depending on the geographical location. The prevalence was higher in the North and Northeast than in the South (Catalan-Soares et al., 2005). Another more recent and large study show Brazilian blood donors prevalence of HTLV-1 on the order of one per 1,000 with regional differences probably due to the ethnic origin of the underlying population with indeed a higher prevalence in donors with black skin color (2.14/1,000) versus mixed (1.58/1,000) or white (0.79/1,000; Carneiro-Proietti et al., 2012). Similarly, HTLV-1 prevalence in pregnant women ranges from 0.1 to 0.8% depending on the tested area (Bittencourt et al., 2001; Olbrich Neto and Meira, 2004; Figueiro-Filho et al., 2007; Ydy et al., 2009; Guimaraes de Souza et al., 2012). Interestingly, Salvador de Bahia, a large city of the eastern part of the country, with a majority of inhabitants of African ancestry, is considered as the Brazilian city with the highest global HTLV-1 prevalence (around 1.3% of blood donors, and 1.8% in the general population; Galvao-Castro et al., 1997; Dourado et al., 2003).

### HTLV-1 in the Caribbean region

This region comprises more than 7,000 islands organized into 30 territories including sovereign states (e.g., Cuba, Jamaica, Haiti and Dominican Republic), overseas departments (e.g., Martinique, Guadeloupe) and dependencies (e.g., Bermuda, Caiman Islands), totalizing around 40 millions of inhabitants. The majority of these islands have a relatively small population (few thousands to half million persons). Only Cuba, Haiti/Dominican Republic, and Jamaica have a population of more than two millions. All the islands, which are mainly peopled with persons of African ancestry, represent a high endemic region for HTLV-1 infection and associated diseases.

Jamaica has been extensively studied concerning HTLV-1 infection (Clark et al., 1985; Murphy et al., 1991). Large series of ATL, TSP/HAM, and infective dermatitis have been reported in this island and in immigrants from Jamaica living, especially, in the United Kingdom (Gibbs et al., 1984; Rodgers-Johnson et al., 1988; Mowbray et al., 1989; La Grenade et al., 1990). In a large cohort of 13,260 Jamaicans from all parts of the island, HTLV-1 seroprevalence was high (mean 6.1%) ranging from 1.7 to 17.4%, depending on sex and age (Murphy et al., 1991). In pregnant women and in blood donors, it was of around 2–3.8% (Wiktor et al., 1993; Dowe et al., 1998; Brady-West and Buchner, 2000; Maloney et al., 2006). The situation is less known but could be quite similar in Haiti and the Dominican Republic. HTLV-1 seroprevalence in pregnant women is around 2.2–4.2% (Allain et al., 1992; Tortevoye et al., 2005) and it is around of 3.8–4.3% in rural Haitian populations (Schill et al., 1989; Grant et al., 1992). Furthermore, some series of ATL and TSP/HAM have been reported mainly in immigrants from this island living in France and the US (Vernant et al., 1987; Bhagavati et al., 1988; Gout et al., 1989; Gessain et al., 1990a; Besson et al., 2001). In the French West Indies (FWI: Martinique and Guadeloupe), HTLV-1 seroprevalence in blood donors is around 0.3–0.4% (Cesaire et al., 1999; Rouet et al., 1999a,b) and in pregnant women from Martinique it is of around 2% (Denis et al., 1988; Mansuy et al., 1999). Furthermore, few hundreds of TSP/HAM and of ATL have been reported during the last 25 years in these islands (Vernant et al., 1987; Plumelle et al., 1993) and in immigrants in Metropolitan France (Gout et al., 1989; Gessain et al., 1990a,c; Besson et al., 2001). HTLV-1 is also endemic at a similar level in Trinidad and Tobago (Blattner et al., 1990; Daisley et al., 1991) as well as in Barbados (Riedel et al., 1989).

In contrast, the studies performed in Cuba indicated that HTLV-1 seroprevalence is very low in this island (Hernandez Ramirez et al., 1991; Silva Cabrera et al., 1997) with also only very few ATL or TSP/HAM cases reported (Estrada et al., 1995). This is very probably linked, partly, to the different ethnic background of the islands with relatively few persons of African ancestry in Cuba as compared to Jamaica, Haiti, or the French West Indies. The estimates of the number of HTLV-1 infected persons for some islands in the Caribbean are given in Table 2.

### HTLV-1 in Asia

Asia is the world’s largest and most populous continent with nearly 3.9 billion people (60% of the world’s current human population). Here we arbitrarily defined four subregions: (1) East Asia (China, Japan, North and South Korea, Mongolia, and Taiwan), (2) North (Siberia), Central (Afghanistan, Iran, Kazakhstan, Kyrgyzstan, Turkmenistan, and Uzbekistan,) and Southwest Asia (Arabian Peninsula, Armenia, Georgia, Israel, Jordan, Lebanon, Syria, and Turkey), (3) South Asia (Bangladesh, Bhutan, India, Nepal, Pakistan, and Sri Lanka,) and (4) Southeast Asia (Brunei, Burma, Cambodia, Indonesia, Laos, Malaysia, Philippines, Singapore, Thailand, East Timor, and Vietnam). Except for some very important endemic foci including mainly Japan and Iran, the prevalence of HTLV-1 and associated diseases in Asia seems very low. However, in most of the areas, due to the lack of large and representative studies, the situation remains still poorly known.

#### East Asia

East Asia comprises more than 1.4 billion people (about 20% of the world population). In Mainland China, no general population-based-study has been performed to our knowledge regarding HTLV-1 prevalence. However, a large-scale study concerning 145,293 blood donors representative of 13 provinces revealed a very low global prevalence (0.013%; Wang et al., 2005). Interestingly, all HTLV-1 seropositive individuals originate from the Fujian province in the Southeast and the prevalence rate in this peculiar province reached 0.055%. This confirmed the prevalence rate (0.06%) previously found in the same province among healthy individuals (Wang et al., 2004). Concerning the HTLV-1 related diseases, while very few cases of TSP/HAM have been reported (Seyfert et al., 2004), some series of ATL cases were found in Chinese patients (Zhuo et al., 1995; Au and Lo, 2005). These ATL cases were mainly distributed in patients from the coastal provinces and the Southeast China especially in the Fujian province.

In Japan, HTLV-1 infection has been extensively studied for more than 30 years. Japan is one of the most important foci of HTLV-1 infection and associated diseases (Watanabe, 2011). Indeed, around 1,000 of ATL cases are diagnosed each year (Tajima, 1990) and several hundreds of TSP/HAM have been reported (Osame et al., 1990), as well as large series of other HTLV-1 associated diseases including uveitis (Mochizuki et al., 1992). The minimal global prevalence of HTLV-1 infection in Japan as determined by screening of blood donors is estimated to be at least 1.08 million in 2006 (Satake et al., 2012). Based on a previous nationwide survey, performed also among blood donors, the number of HTLV-1 infected individuals was around 1.2 million (Tajima, 1990). For several reasons, the prevalence calculated from blood donors survey is very probably lower than the actual value (Satake et al., 2012). Thus, Japan comprises very probably the largest HTLV-1 carriers number in the world. Since the first studies, the repartition of HTLV-1 carriers in Japan is quite uneven with prevalence rates among blood donors varying from 1% in Hokkaido to more than 6% in Kyushu and Okinawa Islands in the Southern part of the archipelago (Hinuma et al., 1982; Maeda et al., 1984; Tajima, 1990). Several studies in pregnant women also revealed such a heterogeneous situation, which origin remains to be determined, with an HTLV-1 prevalence ranging from 0.5% (in North/central Japan) to 5.8% (Southern part of the country; Oki et al., 1992; Umemoto et al., 1994; Goto et al., 1997; Maehama, 2004). In some villages or towns of highly endemic areas, HTLV-1 prevalence can reach 30–40% in adults, aged more than 50 years old (Kohakura et al., 1986; Tajima et al., 1987). Several programs aimed to prevent HTLV-1 transmission from mother to child, by refraining HTLV-1 infected mothers to breast-fed their children, have been implemented with great success (Hino, 2011). Although the HTLV-1 prevalence in Japan is very high, it is not clear why neighboring regions such as East China or Korea have a low prevalence. Indeed, the studies among Korean blood donors from various districts of Korea revealed very low HTLV-1 seropositive rates (0.007–0.25%; Lee et al., 1986; Kim et al., 1999; Kwon et al., 2008). Furthermore, very few cases of ATL and TSP/HAM have been reported in Korean patients (Park et al., 1991; Saito et al., 1993; Jeon et al., 2000).

In Taiwan, slightly more data are available and the prevalence of HTLV-1 infection seems higher. HTLV-1 screening in six blood centers revealed indeed an HTLV-1 seropositivity rate of 0.058% (Lu et al., 2001). Furthermore, in the Taiwanese adult population, aged more than 30 years, the HTLV-1 prevalence varies from 0.82 to 1.63% according to the districts investigated (Chen et al., 1999). Lastly, series of ATL cases have been diagnosed in Taipei (Lee et al., 2010).

#### North, Central, and Southwest Asia

Sporadic HTLV-1 infection cases have been detected in various small ethnic populations of Northern and Eastern Siberia, especially among individuals from the Nivikhi group (Seniuta et al., 1990; Gessain and de The, 1996). These data suggest the possible existence of HTLV-1 foci, which makes impossible a global estimation of the HTLV-1 prevalence in these vast regions. In Central Asia, Iran is by far the most studied country, especially the area of Mashad in the Northeast of the country. According to the type of studies and of tested populations, HTLV-1 prevalence is estimated to range from 0.77 to 3% in adults (Safai et al., 1996; Abbaszadegan et al., 2003; Rafatpanah et al., 2011). A more recent study indicates an HTLV-1 prevalence of 1.66% in adults from the city of Sabzevar located in the Southeast of Mashad and confirmed that HTLV-1 is highly endemic in this region (Azarpazhooh et al., 2012). Furthermore, relatively large series of ATL as well as TSP/HAM cases originating from the Mashad region are reported in Iran (Sidi et al., 1990; Kchour et al., 2009) but also in Iranian immigrants living in European countries, the United States, or Israël (Sidi et al., 1990; Gabarre et al., 1993; Miller et al., 1998). No data are available from other areas of central Asia except scarce findings from Turkmenistan where the HTLV-1 seroprevalence rate among blood donors reached 0.2% (3/1,510; Senyuta et al., 1998).

In Southwest Asia, the HTLV-1 infection appeared to be non-endemic. In Saudi Arabia, there is no case of HTLV-1 infection or extremely low HTLV-1 seroprevalence rates (0.002–0.0052%) reported among blood donors (Bernvil et al., 1997; Arif and Ramia, 1998; El-Hazmi, 2004). The situation is quite comparable in other Persian Gulf countries such as Kuwait where the HTLV-1 seroprevalence observed among blood donors reached 0.009% (Al-Mufti et al., 1997). Nevertheless, few symptomatic HTLV-1 cases have been described in this latter country (Voevodin et al., 1995) and Iraq (Denic et al., 1990).

In Israel, an immigration state, studies indicated that most of infected persons originated from highly endemic countries (Iran, Romania, and Africa). Thus, the HTLV-1 prevalence must be appreciated according to the donors’ countries of origin. However, the HTLV-1 prevalence in blood donors born in Israel reached 0.001% (Stienlauf et al., 2009). Furthermore, some ATL and TSP/HAM cases have been described mainly among immigrants of Romanian or Iranian origin (Sidi et al., 1990; Shtalrid et al., 2005).

In Lebanon, none of 1,900 blood donors screened was seropositive for HTLV-1 (Naman et al., 2002) although one ATL case of Lebanese origin has been described (Bitar et al., 2009). Quite comparable situation is observed in Turkey where none among 10,000 Healthy blood donors was HTLV-1 seroreactive (Sertoz et al., 2010). No data are available, to our knowledge, concerning the HTLV-1 situation in Jordan, Syria, and Armenia.

#### South Asia

India is with China, the second most populated country in the world with 1.2 billion inhabitants. If we consider neighboring countries such as Pakistan, Bangladesh, Sri lanka, and Nepal plus Bhutan and Maldives territories, the South Asian population reached 1.6 billion (a quarter of the world population). In this region, the studies concerning HTLV-1 prevalence are scarce and the most recent one among Indian blood donors revealed a 0.14% prevalence rate (Kumar and Gupta, 2006). Other study demonstrated no serological evidence of HTLV-1 infection among the pregnant women tested (Ramalingam et al., 2001). Despite the low HTLV-1 prevalence rate, some ATL, and TSP/HAM cases have been regularly reported especially in Southern India (Andhra Pradesh, Kerala, and Tamil Nadu; Chandy et al., 1991; Babu et al., 1993; Jain et al., 2008; Ahmed et al., 2012) and Western India (Singhal et al., 1993). Thus, the prevalence of HTLV-1 carriers in India seems rare but taken into account the huge population size, large-scale studies are necessary to assess the global prevalence rate before to conclude that this infection is a minor public health hazard and to estimate a reliable HTLV-1 prevalence rate.

In Bangladesh, despite the large population size, no HTLV-1 prevalence study has been performed. A sporadic occurrence of HTLV-1 infection (0.9%) was found among patients with motor neuron disease called neurolathyrism (Haque et al., 1994). In Pakistan, who shares with Bangladesh the same population size, no data concerning HTLV-1 prevalence is available. This situation is quite the same in others South Asian territories such as Nepal, Sri Lanka, Bhutan, and Maldives archipelago where HTLV-1 infection has never been investigated to our knowledge.

#### Southeast Asia

The Southeast Asian region comprises more than half a billion individuals. However, the situation regarding the HTLV-1 prevalence in the most populated countries has been poorly described. In Indonesia, the few studies revealed no HTLV-1 carrier among the blood donors tested (Tanggo et al., 2000). In Philippines, anecdotal, and ancient study suggested that the infection is present in the general population (Ishida et al., 1988). In the neighboring countries such as Malaysia, Vietnam, Cambodia, Laos, and Burma, no reliable information is available concerning the HTLV-1 global epidemiology. Moreover, no evidence of HTLV-1 infection among Hmong individuals from Northern Thailand was reported (Louisiriotchanakul et al., 2000) and a study performed on Hmong refugees living in French Guyana confirmed this data (Tortevoye et al., 2000). The situation seems quite different in the eastern part of New Guinea Island called Irian Jaya, where sporadic cases of HTLV-1 infection have been reported in some small isolated tribes (Re et al., 1989). Despite the lack of population-based studies in the Southeast Asian populations, the HTLV-1 prevalence rate seems to be low.

### HTLV-1 in Oceania

Oceania is a vast zone between continental Asia and the Americas comprising mostly coral atolls and volcanic islands. It is divided into three subregions, Micronesia (Kiribati, Marshall Islands, and Nauru), Australo-Melanesia (Australia, Fiji Islands, New Caledonia, Papua New Guinea, Solomon Islands, and Vanuatu), and Polynesia (American Samoa, Cook Islands, Easter Island, French Polynesia, Hawaii, New Zealand, Samoa, Tonga, Tuvalu, Wallis, and Futuna). The population is around 35 millions individuals with two third of Australian origin.

#### Australo-Melanesia

Only few population-based-study using solid serological confirmation criteria have been performed regarding HTLV-1 prevalence in the Australo-Melanesian region. Interestingly, few studies, conducted among Papuan aboriginal populations, demonstrated that some remote tribes as the Hagahai population living in the highlands (Madang province), exhibited higher HTLV-1 prevalence rate than population groups originating from different Papuan provinces such as the Sepik province (Yanagihara et al., 1990; Sanders et al., 1993; Yamaguchi et al., 1993; Takao et al., 2000). In the Solomon Islands, the HTLV-1 seroprevalence rate range from 1.2 to 3% according to the populations (hospitalized individuals, blood donors) and the islands investigated (Yanagihara et al., 1991; Nicholson et al., 1992; Furusyo et al., 1999). In Vanuatu archipelago, a large-scale population-based-study (4,247 individuals mostly adults) showed that the HTLV-1 prevalence rate was of 0.62% (Cassar et al., 2007). In Fiji and New Caledonia archipelagoes, no HTLV-1 infection was brought out by the very few studies performed among the native-born populations tested (Louis et al., 1990; Nicholson et al., 1992; Chungue et al., 1993).

In Australia, the situation for HTLV-1 is quite different as we considered Aboriginal populations from Central Australia or blood donors comprising mainly white non-Aboriginal individuals living in coastal areas. Thus, the studies conducted among blood donors from main Australian cities (Adelaide, Brisbane, Melbourne, Perth, and Sydney) revealed that HTLV-1 prevalence rate is very low, comprised between 0.001 and 0.032% (Whyte, 1997; Polizzotto et al., 2008). In contrast, several studies indicated that the Aboriginal groups of inland Australian regions represent a high HTLV-1 endemic population (May et al., 1988; Einsiedel et al., 2010, 2012). Moreover, HTLV-1 related cases, ATL and/or TSP/HAM, have been described among the Aboriginal communities of central Australia (Kirkland et al., 1991; Rajabalendaran et al., 1993), Papua New Guinea (Yanagihara et al., 1990), and the Solomon Islands (Ajdukiewicz et al., 1989).

#### Polynesia/Micronesia

The few reliable studies performed in Polynesia/Micronesia regarding the HTLV-1 prevalence among native-born Polynesian individuals, indicated that the infection is absent or very rare (Nicholson et al., 1992; Chungue et al., 1993; Ohkura et al., 1999). In the Hawaiian archipelago, the virus is present but the studies suggested that HTLV-1 was introduced with the Japanese immigration (Kimata et al., 1989). Indeed, ATL and TSP/HAM cases were observed among immigrants from Southern Japan and their descendants (Yim et al., 1986; Yanagihara et al., 1989; Dixon et al., 1990). Few other studies have described the HTLV-1 situation in the Cook Islands, Kiribati, New Zealand, the Samoa and Wallis, and Futuna, but no HTLV-1 seropositive case has been documented to date (Reddy et al., 1987; Louis et al., 1990; Nicholson et al., 1992; Chungue et al., 1993).

## Conclusion

Our best estimates range approximately from 5 to 10 millions HTLV-1 infected individuals. However, these results were only based on nearly 1.5 billion of individuals originating from known HTLV-1 endemic areas with reliable available epidemiological data. Correct estimates in other highly populated regions, such as China, India, the Maghreb, and East Africa, is currently not possible. The real number of HTLV-1 carriers is thus very probably much higher.

## Conflict of Interest Statement

The authors declare that the research was conducted in the absence of any commercial or financial relationships that could be construed as a potential conflict of interest.

## References

[B1] Abbaszadegan M. R. Gholamin M. Tabatabaee A. Farid R. Houshmand M. Abbaszadegan M. (2003). Prevalence of human T-lymphotropic virus type 1 among blood donors from Mashhad, Iran. J. Clin. Microbiol. 41, 2593–2595 10.1128/JCM.41.6.2593-2595.2003 12791885 PMC156501

[B2] Ades A. E. Parker S. Walker J. Edginton M. Taylor G. P. Weber J. N. (2000). Human T cell leukaemia/lymphoma virus infection in pregnant women in the United Kingdom: population study. BMJ 320, 1497–1501 10.1136/bmj.320.7248.1497 10834889 PMC27390

[B3] Ahmed F. Murthy S. S. Mohan M. V. Rajappa S. J. (2012). HTLV 1 associated adult T cell lymphoma/leukemia a clinicopathologic, immunophenotypic tale of three cases from non-endemic region of south India. Indian J. Pathol. Microbiol. 55, 92–96 10.4103/0377-4929.94870 22499311

[B4] Ajdukiewicz A. Yanagihara R. Garruto R. M. Gajdusek D. C. Alexander S. S. (1989). HTLV-1 myeloneuropathy in the Solomon Islands. N. Engl. J. Med. 321, 615–616 10.1056/NEJM198908313210914 2548101

[B5] Alarcon J. O. Friedman H. B. Montano S. M. Zunt J. R. Holmes K. K. Quinnan G. V. Jr. (2006). High endemicity of human T-cell lymphotropic virus type 1 among pregnant women in peru. J. Acquir. Immune Defic. Syndr. 42, 604–609 10.1097/01.qai.0000221680.52563.d5 16773029 PMC2683844

[B6] Allain J. P. Hodges W. Einstein M. H. Geisler J. Neilly C. Delaney S. (1992). Antibody to HIV-1, HTLV-I, and HCV in three populations of rural Haitians. J. Acquir. Immune Defic. Syndr. 5, 1230–1236 1333530

[B7] Al-Mufti S. Voevodin A. Ahmed S. Al-Hamdan S. Al-Bisher A. A. (1997). Prevalence of human T-cell lymphotropic virus type I infection among volunteer blood donors in Kuwait. J. Acquir. Immune Defic. Syndr. Hum. Retrovirol. 15, 88–90 10.1097/00042560-199705010-00018 9215663

[B8] Ampofo W. Nii-Trebi N. Ansah J. Abe K. Naito H. Aidoo S. (2002). Prevalence of blood-borne infectious diseases in blood donors in Ghana. J. Clin. Microbiol. 40, 3523–3525 10.1128/JCM.40.9.3523-3525.2002 12202610 PMC130790

[B9] Analo H. I. Akanmu A. S. Akinsete I. Njoku O. S. Okany C. C. (1998). Seroprevalence study of HTLV-1 and HIV infection in blood donors and patients with lymphoid malignancies in Lagos, Nigeria. Cent. Afr. J. Med. 44, 130–134 9810411

[B10] Andersson S. Dias F. Mendez P. J. Rodrigues A. Biberfeld G. (1997). HTLV-I and -II infections in a nationwide survey of pregnant women in Guinea-Bissau, West Africa. J. Acquir. Immune Defic. Syndr. Hum. Retrovirol. 15, 320–322 10.1097/00042560-199708010-00014 9292595

[B11] Ando Y. Nakano S. Saito K. Shimamoto I. Ichijo M. Toyama T. (1987). Transmission of adult T-cell leukemia retrovirus (HTLV-I) from mother to child: comparison of bottle- with breast-fed babies. Jpn. J. Cancer Res. 78, 322–324 2884205

[B12] Arango C. Concha M. Zaninovic V. Corral R. Biojo R. Borrero I. (1988). Epidemiology of tropical spastic paraparesis in Columbia and associated HTLV-I infection. Ann. Neurol. 23(Suppl.), S161–S165 10.1002/ana.410230736 2894809

[B13] Araujo A. Q. Andrade-Filho A. S. Castro-Costa C. M. Menna-Barreto M. Almeida S. M. (1998). HTLV-I-associated myelopathy/tropical spastic paraparesis in Brazil: a nationwide survey. HAM/TSP Brazilian Study Group. J. Acquir. Immune Defic. Syndr. Hum. Retrovirol. 19, 536–541 10.1097/00042560-199812150-00014 9859969

[B14] Arif M. Ramia S. (1998). Seroprevalence of human T-lymphotropic virus type I (HTLV-I) in Saudi Arabia. Ann. Trop. Med. Parasitol. 92, 305–309 10.1080/00034989859870 9713546

[B15] Armah H. B. Narter-Olaga E. G. Adjei A. A. Asomaning K. Gyasi R. K. Tettey Y. (2006). Seroprevalence of human T-cell lymphotropic virus type I among pregnant women in Accra, Ghana. J. Med. Microbiol. 55, 765–770 10.1099/jmm.0.46426-0 16687597

[B16] Au W. Y. Lo J. Y. (2005). HTLV-1-related lymphoma in Hong Kong Chinese. Am. J. Hematol. 78, 80–81 10.1002/ajh.20217 15609285

[B17] Azarpazhooh M. R. Hasanpour K. Ghanbari M. Rezaee S. A. Mashkani B. Hedayati-Moghaddam M. R. (2012). Human T-lymphotropic virus type 1 prevalence in northeastern Iran, Sabzevar: an epidemiologic-based study and phylogenetic Analysis. AIDS Res. Hum. Retroviruses 28, 1095–1101 22229796 10.1089/AID.2011.0248

[B18] Babu P. G. Gnanamuthu C. Saraswathi N. K. Nerurkar V. R. Yanagihara R. John T. J. (1993). HTLV-I-associated myelopathy in south India. AIDS Res. Hum. Retroviruses 9, 499–500 10.1089/aid.1993.9.499 8347394

[B19] Becker W. B. Becker M. L. Homma T. Brede H. D. Kurth R. (1985). Serum antibodies to human T-cell leukaemia virus type I in different ethnic groups and in non-human primates in South Africa. S. Afr. Med. J. 67, 445–449 2984793

[B20] Beltran B. Quinones P. Morales D. Cotrina E. Castillo J. J. (2011). Different prognostic factors for survival in acute and lymphomatous adult T-cell leukemia/lymphoma. Leuk. Res. 35, 334–339 10.1016/j.leukres.2010.08.006 20828817

[B21] Berini C. A. Gendler S. A. Pascuccio S. Eirin M. E. McFarland W. Page K. (2010). Decreasing trends in HTLV-1/2 but stable HIV-1 infection among replacement donors in Argentina. J. Med. Virol. 82, 873–877 10.1002/jmv.21728 20336721

[B22] Bernvil S. S. Andrews V. Coulter N. (1997). International forum: Saudi Arabia. Donor screening for HTLV-I in Saudi Arabia: is it cost effective? Transfus. Sci. 18, 45–47 10.1016/S0955-3886(96)00076-8 10174291

[B23] Bertherat E. Makuwa M. Renaut A. Nabias R. Georges-Courbot M. C. (1998). HIV-1, HTLV-I, and HTLV-II in a semiurban population in East Gabon. J. Acquir. Immune Defic. Syndr. Hum. Retrovirol. 19, 430–432 10.1097/00042560-199812010-00017 9833756

[B24] Besson C. Gonin C. Brebion A. Delaunay C. Panelatti G. Plumelle Y. (2001). Incidence of hematological malignancies in Martinique, French West Indies, overrepresentation of multiple myeloma and adult T cell leukemia/lymphoma. Leukemia 15, 828–831 10.1038/sj.leu.2402040 11368445

[B25] Bhagavati S. Ehrlich G. Kula R. W. Kwok S. Sninsky J. Udani V. (1988). Detection of human T-cell lymphoma/leukemia virus type I DNA and antigen in spinal fluid and blood of patients with chronic progressive myelopathy. N. Engl. J. Med. 318, 1141–1147 10.1056/NEJM198805053181801 2896300

[B26] Bhatt N. B. Gudo E. S. Sema C. Bila D. Di Mattei P. Augusto O. (2009). Loss of correlation between HIV viral load and CD4+ T-cell counts in HIV/HTLV-1 co-infection in treatment naive Mozambican patients. Int. J. STD AIDS 20, 863–868 10.1258/ijsa.2008.008401 19948902

[B27] Bhigjee A. I. Kelbe C. Haribhai H. C. Windsor I. M. Hoffmann M. H. Modi G. (1990). Myelopathy associated with human T cell lymphotropic virus type I (HTLV-I) in natal, South Africa. A clinical and investigative study in 24 patients. Brain 113(Pt 5), 1307–1320 10.1093/brain/113.5.1307 2245298

[B28] Bhigjee A. I. Thaler D. Madurai S. Gouws E. Bill P. L. (1994). Seroprevalence of HTLV-I in Natal/KwaZulu. S. Afr. Med. J. 84, 368 7740402

[B29] Bhigjee A. I. Vinsen C. Windsor I. M. Gouws E. Bill P. L. Tait D. (1993). Prevalence and transmission of HTLV-I infection in Natal/KwaZulu. S. Afr. Med. J. 83, 665–667 8123179

[B30] Biggar R. J. Neequaye J. E. Neequaye A. R. Ankra-Badu G. A. Levine P. H. Manns A. (1993). The prevalence of antibodies to the human T lymphotropic virus (HTLV) in Ghana, West Africa. AIDS Res. Hum. Retroviruses 9, 505–511 10.1089/aid.1993.9.505 8347395

[B31] Biggar R. J. Saxinger C. Gardiner C. Collins W. E. Levine P. H. Clark J. W. (1984). Type-I HTLV antibody in urban and rural Ghana, West Africa. Int. J. Cancer 34, 215–219 10.1002/ijc.2910340212 6088402

[B32] Biglione M. M. Astarloa L. Salomon H. E. (2005). High prevalence of HTLV-I and HTLV-II among blood donors in Argentina: a South American health concern. AIDS Res. Hum. Retroviruses 21, 1–4 10.1089/aid.2005.21.1 15665638

[B33] Biglione M. M. Pizarro M. Puca A. Salomon H. E. Berria M. I. (2003). A cluster of human T-cell lymphotropic virus type I-associated myelopathy/tropical spastic paraparesis in Jujuy, Argentina. J. Acquir. Immune Defic. Syndr. 32, 441–445 10.1097/00126334-200304010-00015 12640204

[B34] Bitar N. Hajj H. E. Houmani Z. Sabbah A. Otrock Z. K. Mahfouz R. (2009). Adult T-cell leukemia/lymphoma in the Middle East: first report of two cases from Lebanon. Transfusion 49, 1859–1864 10.1111/j.1537-2995.2009.02223.x 19453978

[B35] Bittencourt A. L. Barbosa H. S. Vieira M. D. Farre L. (2009). Adult T-cell leukemia/lymphoma (ATL) presenting in the skin: clinical, histological and immunohistochemical features of 52 cases. Acta Oncol. 48, 598–604 10.1080/02841860802657235 19165640

[B36] Bittencourt A. L. Dourado I. Filho P. B. Santos M. Valadao E. Alcantara L. C. (2001). Human T-cell lymphotropic virus type 1 infection among pregnant women in northeastern Brazil. J. Acquir. Immune Defic. Syndr. 26, 490–494 10.1097/00126334-200104150-00016 11391171

[B37] Blakeslee J. R. Jr. McClure H. M. Anderson D. C. Bauer R. M. Huff L. Y. Olsen R. G. (1987). Chronic fatal disease in gorillas seropositive for simian T-lymphotropic virus I antibodies. Cancer Lett. 37, 1–6 10.1016/0304-3835(87)90139-X 2822228

[B38] Blank A. Yamaguchi K. Blank M. Zaninovic V. Sonoda S. Takatsuki K. (1993). Six Colombian patients with adult T-cell leukemia/lymphoma. Leuk. Lymphoma 9, 407–412 10.3109/10428199309148542 8348076

[B39] Blattner W. A. Nomura A. Clark J. W. Ho G. Y. Nakao Y. Gallo R. (1986). Modes of transmission and evidence for viral latency from studies of human T-cell lymphotrophic virus type I in Japanese migrant populations in Hawaii. Proc. Natl. Acad. Sci. U.S.A. 83, 4895–4898 10.1073/pnas.83.13.4895 3014518 PMC323850

[B40] Blattner W. A. Saxinger C. Riedel D. Hull B. Taylor G. Cleghorn F. (1990). A study of HTLV-I and its associated risk factors in Trinidad and Tobago. J. Acquir. Immune Defic. Syndr. 3, 1102–1108 2213511

[B41] Blayney D. W. Jaffe E. S. Blattner W. A. Cossman J. Robert-Guroff M. Longo D. L. (1983). The human T-cell leukemia/lymphoma virus associated with American adult T-cell leukemia/lymphoma. Blood 62, 401–405 6223675

[B42] Brady-West D. C. Buchner L. M. (2000). Retrospective audit of blood donation at a hospital-based blood centre. Implications for blood product supply and safety. West Indian Med. J. 49, 226–228 11076215

[B43] Brant L. J. Cawley C. Davison K. L. Taylor G. P. (2011). Recruiting individuals into the HTLV cohort study in the United Kingdom: clinical findings and challenges in the first six years, 2003 to 2009. Euro Surveill. 16, pii=20017. 22115046 10.2807/ese.16.46.20017-en

[B44] Cabrera M. E. Gray A. M. Cartier L. Araya F. Hirsh T. Ford A. M. (1991). Simultaneous adult T-cell leukemia/lymphoma and sub-acute polyneuropathy in a patient from Chile. Leukemia 5, 350–353 1851242

[B45] Calderon E. J. Rey C. Medrano F. J. Sanchez-Roman J. Soriano V. Torres Y. (1995). Prevalence of infection by human T-cell leukemia virus types I and II in southern Spain. Eur. J. Clin. Microbiol. Infect. Dis. 14, 686–690 10.1007/BF01690875 8565986

[B46] Cantor K. P. Weiss S. H. Goedert J. J. Battjes R. J. (1991). HTLV-I/II seroprevalence and HIV/HTLV coinfection among U.S. intravenous drug users. J. Acquir. Immune Defic. Syndr. 4, 460–467 2016683

[B47] Carles G. Tortevoye P. Tuppin P. Ureta-Vidal A. Peneau C. El Guindi W. (2004). HTLV1 infection and pregnancy. J. Gynecol. Obstet. Biol. Reprod. (Paris) 33, 14–20 10.1016/S0368-2315(04)96307-7 14968050

[B48] Carneiro-Proietti A. B. Sabino E. C. Leao S. Salles N. A. Loureiro P. Sarr M. (2012). Human T-lymphotropic virus type 1 and type 2 seroprevalence, incidence, and residual transfusion risk among blood donors in Brazil during 2007–2009. AIDS Res. Hum. Retroviruses 28, 1265–1272 10.1089/aid.2011.0143 22324906 PMC3448098

[B49] Cartier L. Araya F. Castillo J. L. Ruiz F. Gormaz A. Tajima K. (1992). Progressive spastic paraparesis associated with human T-cell leukemia virus type I (HTLV-I). Intern. Med. 31, 1257–1261 10.2169/internalmedicine.31.1257 1295619

[B50] Cartier L. Mora C. Araya F. Castillo J. Verdugo R. Miller M. (1989). HTLV-I positive spastic paraparesis in a temperate zone. Lancet 1, 556 10.1016/S0140-6736(89)90098-6 2564088

[B51] Cassar O. Capuano C. Bassot S. Charavay F. Duprez R. Afonso P. V. (2007). Human T lymphotropic virus type 1 subtype C melanesian genetic variants of the Vanuatu Archipelago and Solomon Islands share a common ancestor. J. Infect. Dis. 196, 510–521 10.1086/519167 17624835

[B52] Catalan-Soares B. Carneiro-Proietti A. B. Proietti F. A. (2005). Heterogeneous geographic distribution of human T-cell lymphotropic viruses I and II (HTLV-I/II): serological screening prevalence rates in blood donors from large urban areas in Brazil. Cad. Saude Publica 21, 926–931 10.1590/S0102-311X2005000300027 15868051

[B53] Caterino-de-Araujo A. Magri M. C. Costa E. A. Manuel R. C. (2010). Prevalence of human T-cell lymphotropic virus (HTLV-1/2) in individuals from public health centers in Mozambique. AIDS Res. Hum. Retroviruses 26, 559–561 10.1089/aid.2009.0269 20438381

[B54] Catovsky D. Greaves M. F. Rose M. Galton D. A. Goolden A. W. McCluskey D. R. (1982). Adult T-cell lymphoma-leukaemia in Blacks from the West Indies. Lancet 1, 639–643 10.1016/S0140-6736(82)92200-0 6121963

[B55] Ceesay M. M. Matutes E. Taylor G. P. Fields P. Cavenagh J. Simpson S. (2012). Phase II study on combination therapy with CHOP-Zenapax for HTLV-I associated adult T-cell leukaemia/lymphoma (ATLL). Leuk. Res. 36, 857–861 10.1016/j.leukres.2011.12.004 22209076

[B56] Cesaire R. Bera O. Maier H. Lezin A. Martial J. Ouka M. (1999). Seroin determinate patterns and seroconversions to human T-lymphotropic virus type I positivity in blood donors from Martinique, French West Indies. Transfusion 39, 1145–1149 10.1046/j.1537-2995.1999.39101145.x 10532611

[B57] Chandy M. Babu P. G. Saraswathy N. K. Ishida T. John T. J. (1991). HTLV-1 infection in patients with leukaemia in southern India. Lancet 338, 380–381 10.1016/0140-6736(91)90510-V 1677713

[B58] Chavance M. Frery N. Valette I. Monplaisir N. Schaffar L. (1989). Cohort effect of HTLV-I seroprevalence in southern Japan. Lancet 2, 1337 10.1016/S0140-6736(89)91944-2 2574285

[B59] Chen Y. M. Ting S. T. Lee C. M. Liu W. T. Pan W. H. Cheng A. T. (1999). Community-based molecular epidemiology of HTLV type I in Taiwan and Kinmen: implication of the origin of the cosmopolitan subtype in northeast Asia. AIDS Res. Hum. Retroviruses 15, 229–237 10.1089/088922299311691 10052753

[B60] Chiavetta J. Nusbacher J. Tam F. Wall A. Steaffens J. Lee H. (1992). Prevalence of antibody to human T-cell lymphotropic virus type I/II in people of Caribbean origin in Toronto. CMAJ 147, 1493–1498 1423089 PMC1336545

[B61] Chironna M. Calabro M. L. Quarto M. Germinario C. Fiore J. R. Favero A. (1996). HTLV-I and HTLV-II infections in subjects at risk for HIV-I infection from southeastern Italy (Apulia region). Int. J. Cancer 65, 746–750 10.1002/(SICI)1097-0215(19960315)65:6<746::AID-IJC6>3.0.CO;2-Z 8631585

[B62] Chungue E. Boutin J. P. Le Marchand L. Philippon G. Le Guellec A. Chanteau S. (1993). Seroepidemiological survey of HTLV-I infection in French Polynesia, Cook Islands and Fiji. Eur. J. Epidemiol. 9, 347–350 10.1007/BF00146276 8405324

[B63] Clark J. Saxinger C. Gibbs W. N. Lofters W. Lagranade L. Deceulaer K. (1985). Seroepidemiologic studies of human T-cell leukemia/lymphoma virus type I in Jamaica. Int. J. Cancer 36, 37–41 10.1002/ijc.2910360209 2862109

[B64] Cnudde F. Gessain A. Dandelot J. B. Carlier P. Julvez J. Andriambao P. S. (1991). HTLV-I in neurological patients from some Indian Ocean islands. J. Acquir. Immune Defic. Syndr. 4, 734–735 2051310

[B65] Collenberg E. Ouedraogo T. Ganame J. Fickenscher H. Kynast-Wolf G. Becher H. (2006). Seroprevalence of six different viruses among pregnant women and blood donors in rural and urban Burkina Faso: a comparative analysis. J. Med. Virol. 78, 683–692 10.1002/jmv.20593 16555290

[B66] Courouce A. M. Pillonel J. Lemaire J. M. Maniez M. Brunet J. B. (1993). Seroepidemiology of HTLV-I/II in universal screening of blood donations in France. AIDS 7, 841–847 10.1097/00002030-199306000-00013 8103342

[B67] Courtois F. Barin F. Larsen M. Brossard Y. Masselin A. Engelman P. (1990). HTLV-I/II infection in pregnant women in Paris. Lancet 335, 1103 10.1016/0140-6736(90)92681-7 1970406

[B68] Cunha L. Plouzeau C. Ingrand P. Gudo J. P. Ingrand I. Mondlane J. (2007). Use of replacement blood donors to study the epidemiology of major blood-borne viruses in the general population of Maputo, Mozambique. J. Med. Virol. 79, 1832–1840 10.1002/jmv.21010 17935167

[B69] da Silva Z. J. Nielsen J. Andersen A. Oliveira I. Dias F. Rodrigues A. (2009). Decline in human T-cell lymphotropic virus-1 prevalence in urban areas of Bissau, Guinea-Bissau: exploring the association with HIV infections. AIDS 23, 637–639 10.1097/QAD.0b013e32832403e8 19242315

[B70] Daisley H. Charles W. Landeau P. Jackman L. Batson M. Gomez-Adams K. (1991). Screening for HTLV-1 in healthy blood donors in Trinidad and Tobago, West Indies. Trop. Med. Parasitol. 42, 404–406 1796241

[B71] Dalekos G. N. Zervou E. Karabini F. Elisaf M. Bourantas K. Siamopoulos K. C. (1995). Prevalence of antibodies to human T-lymphotropic virus types I and II in volunteer blood donors and high-risk groups in northwestern Greece. Transfusion 35, 503–506 10.1046/j.1537-2995.1995.35695288770.x 7770902

[B72] Davidson F. Lycett C. Jarvis L. M. Kerr D. Lumley S. Petrik J. (2006). Detection of HTLV-I and -II in Scottish blood donor samples and archive donations. Vox Sang. 91, 231–236 10.1111/j.1423-0410.2006.00816.x 16958835

[B73] de Oliveira M. S. Matutes E. Famadas L. C. Schulz T. F. Calabro M. L. Nucci M. (1990). Adult T-cell leukaemia/lymphoma in Brazil and its relation to HTLV-I. Lancet 336, 987–990 10.1016/0140-6736(90)92432-H 1977015

[B74] de Rivera I. L. Amador L. Mourra S. Li Z. Rasheed S. (1995). Geographical clustering of human T-cell lymphotropic virus type 1 infection in Honduras. J. Clin. Microbiol. 33, 2999–3003 8576361 10.1128/jcm.33.11.2999-3003.1995 PMC228622

[B75] de The G. Bomford R. (1993). An HTLV-I vaccine: why, how, for whom? AIDS Res. Hum. Retroviruses 9, 381–386 10.1089/aid.1993.9.381 8318266

[B76] de The G. Gessain A. (1986). Seroepidemiologic data on viral infections (HTLV-I and LAV/HTLV-III) in the Caribbean region and intertropical Africa. Ann. Pathol. 6, 261–264 2880599

[B77] Dekaban G. A. Oger J. J. Foti D. King E. E. Waters D. J. Picard F. J. (1994). HTLV-I infection associated with disease in aboriginal Indians from British Columbia: a serological and PCR analysis. Clin. Diagn. Virol. 2, 67–78 10.1016/0928-0197(94)90039-6 15566754

[B78] Del Mistro A. Chotard J. Hall A. J. Fortuin M. Whittle H. De Rossi A. (1994). HTLV-I/II seroprevalence in the Gambia: a study of mother-child pairs. AIDS Res. Hum. Retroviruses 10, 617–620 10.1089/aid.1994.10.617 7917523

[B79] Delaporte E. Buve A. Nzila N. Goeman J. Dazza M. C. Henzel D. (1995). HTLV-I infection among prostitutes and pregnant women in Kinshasa, Zaire: how important is high-risk sexual behavior? J. Acquir. Immune Defic. Syndr. Hum. Retrovirol. 8, 511–515 10.1097/00042560-199504120-00012 7697449

[B80] Delaporte E. Dupont A. Peeters M. Josse R. Merlin M. Schrijvers D. (1988). Epidemiology of HTLV-I in Gabon (Western Equatorial Africa). Int. J. Cancer 42, 687–689 10.1002/ijc.2910420509 3182104

[B81] Delaporte E. Monplaisir N. Louwagie J. Peeters M. Martin-Prevel Y. Louis J. P. (1991). Prevalence of HTLV-I and HTLV-II infection in Gabon, Africa: comparison of the serological and PCR results. Int. J. Cancer 49, 373–376 10.1002/ijc.2910490310 1917135

[B82] Delaporte E. Peeters M. Durand J. P. Dupont A. Schrijvers D. Bedjabaga L. (1989a). Seroepidemiological survey of HTLV-I infection among randomized populations of western central African countries. J. Acquir. Immune Defic. Syndr. 2, 410–413 2754613

[B83] Delaporte E. Peeters M. Simoni M. Piot P. (1989b). HTLV-I infection in western equatorial Africa. Lancet 2, 1226 10.1016/S0140-6736(89)91840-0 2572942

[B84] Denic S. Abramson J. Anandakrishnan R. Krishnamurthy M. Dosik H. (1988). The first report of familial adult T-cell leukemia lymphoma in the United States. Am. J. Hematol. 27, 281–283 10.1002/ajh.2830270410 2895582

[B85] Denic S. Nolan P. Doherty J. Garson J. Tuke P. Tedder R. (1990). HTLV-I infection in Iraq. Lancet 336, 1135–1136 10.1016/0140-6736(90)92618-R 1978016

[B86] Denis F. Verdier M. Chout R. Ramiandrisoa H. Sangare A. Prince-David M. (1988). Prevalence of HTLV-1 virus in pregnant women in Black Africa, Martinique, and foreigners living in France. Bull. Acad. Natl. Med. 172, 717–722 3056585

[B87] de-The G. Gessain A. Gazzolo L. Robert-Guroff M. Najberg G. Calender A. (1985). Comparative seroepidemiology of HTLV-I and HTLV-III in the French West Indies and some African countries. Cancer Res. 45, 4633s–4636s 2990698

[B88] Diop S. Calattini S. Abah-Dakou J. Thiam D. Diakhate L. Gessain A. (2006). Seroprevalence and molecular epidemiology of human T-Cell leukemia virus type 1 (HTLV-1) and HTLV-2 in blood donors from Dakar, Senegal. J. Clin. Microbiol. 44, 1550–1554 10.1128/JCM.44.4.1550-1554.2006 16597891 PMC1448682

[B89] Dixon P. S. Bodner A. J. Okihiro M. Milbourne A. Diwan A. Nakamura J. M. (1990). Human T-lymphotropic virus type I (HTLV-I) and tropical spastic paraparesis or HTLV-I-associated myelopathy in Hawaii. West. J. Med. 152, 261–267 2139754 PMC1002326

[B90] Dosik H. Denic S. Patel N. Krishnamurthy M. Levine P. H. Clark J. W. (1988). Adult T-cell leukemia/lymphoma in Brooklyn. JAMA 259, 2255–2257 10.1001/jama.259.15.2255 2895192

[B91] Dougan S. Smith A. Tosswill J. C. Davison K. Zuckerman M. Taylor G. P. (2005). New diagnoses of HTLV infection in England and Wales: 2002–2004. Euro Surveill. 10, 232–235 16282645

[B92] Dourado I. Alcantara L. C. Barreto M. L. da Gloria Teixeira M. Galvao-Castro B. (2003). HTLV-I in the general population of Salvador, Brazil: a city with African ethnic and sociodemographic characteristics. J. Acquir. Immune Defic. Syndr. 34, 527–531 10.1097/00126334-200312150-00013 14657765

[B93] Dowe G. King S. D. Smikle M. F. Wynter H. H. Chout R. Klaskala W. (1998). Prevalence of viral and bacterial sexually transmitted pathogens in Jamaican pregnant women. West Indian Med. J. 47, 23–25 9619092

[B94] Dumas M. Houinato D. Verdier M. Zohoun T. Josse R. Bonis J. (1991). Seroepidemiology of human T-cell lymphotropic virus type I/II in Benin (West Africa). AIDS Res. Hum. Retroviruses 7, 447–451 10.1089/aid.1991.7.447 1873079

[B95] Duval A. Rivet J. Moulonguet I. Cassar O. Agbalika F. Wallach D. (2010). Atypical presentation of adult T-cell leukaemia/lymphoma due to HTLV-1: prurigo nodularis lasting twelve years followed by an acute micropapular eruption. Acta Derm. Venereol. 90, 287–290 20526548 10.2340/00015555-0846

[B96] Eguchi K. Fujii H. Oshima K. Otani M. Matsuo T. Yamamoto T. (2009). Human T-lymphotropic virus type 1 (HTLV-1) genetic typing in Kakeroma Island, an island at the crossroads of the ryukyuans and Wajin in Japan, providing further insights into the origin of the virus in Japan. J. Med. Virol. 81, 1450–1456 10.1002/jmv.21540 19551824

[B97] Einsiedel L. Fernandes L. Spelman T. Steinfort D. Gotuzzo E. (2012). Bronchiectasis is associated with human T-lymphotropic virus 1 infection in an Indigenous Australian population. Clin. Infect. Dis. 54, 43–50 10.1093/cid/cir766 22095566

[B98] Einsiedel L. Verdonck K. Gotuzzo E. (2010). “Human T-lymphotropic virus 1: clinical aspects of a neglected infection among indigenous populations,” in Emerging Infections, Vol. 9, eds Scheld M. W. Grayson L. M. Hughes M. J. (Washington, DC: ASM Press), 109–127

[B99] el-Farrash M. A. Badr M. F. Hawas S. A. el-Nashar N. M. Imai J. Komoda H. (1988). Sporadic carriers of human T-lymphotropic virus type I in northern Egypt. Microbiol. Immunol. 32, 981–984 3062330 10.1111/j.1348-0421.1988.tb01461.x

[B100] El-ghazzawi E. Hunsmann G. Schneider J. (1987). Low prevalence of antibodies to HIV-1 and HTLV-I in Alexandria, Egypt. AIDS Forsch. 2, 639 12342620

[B101] El-Hazmi M. M. (2004). Prevalence of HBV, HCV, HIV-1, 2 and HTLV-I/II infections among blood donors in a teaching hospital in the Central region of Saudi Arabia. Saudi Med. J. 25, 26–33 14758374

[B102] Engelbrecht S. Koulinska I. Smith T. L. Robson B. A. Barreto J. van Rensburg E. J. (1999). Subtyping of human T cell lymphotropic virus type I from tropical spastic paraparesis/HTLV-associated myelopathy patients in Mozambique. AIDS Res. Hum. Retroviruses 15, 71–72 10.1089/088922299311286 10024055

[B103] Estrada R. A. Luis S. Mustelier R. Ruiz W. Rodriguez B. Miranda A. (1995). Absence of human retroviral antibodies in epidemic neuropathy in Cuba: report of the first two cases of HTLV-I-associated tropical spastic paraparesis observed in Cuba. J. Neurol. Sci. 128, 112–113 10.1016/0022-510X(94)00247-L 7722529

[B104] Etenna S. L. Caron M. Besson G. Makuwa M. Gessain A. Mahe A. (2008). New insights into prevalence, genetic diversity, and proviral load of human T-cell leukemia virus types 1 and 2 in pregnant women in Gabon in equatorial central Africa. J. Clin. Microbiol. 46, 3607–3614 10.1128/JCM.01249-08 18845819 PMC2576568

[B105] Ferrante P. Mancuso R. Zuffolato R. Puricelli S. Mannella E. Romano L. (1997). Molecular analysis of HTLV-I and HTLV-II isolates from Italian blood donors, intravenous drug users and prisoners. New Microbiol. 20, 93–104 9208419

[B106] Figueiro-Filho E. A. Senefonte F. R. Lopes A. H. de Morais O. O. Souza Junior V. G. Maia T. L. (2007). Frequency of HIV-1, rubella, syphilis, toxoplasmosis, cytomegalovirus, simple herpes virus, hepatitis B, hepatitis C, Chagas disease and HTLV I/II infection in pregnant women of State of Mato Grosso do Sul. Rev. Soc. Bras. Med. Trop. 40, 181–187 10.1590/S0037-86822007000200007 17568885

[B107] Filippone C. Bassot S. Betsem E. Tortevoye P. Guillotte M. Mercereau-Puijalon O. (2012). A new and frequent human T-cell leukemia virus indeterminate Western blot pattern: epidemiological determinants and PCR results in central African inhabitants. J. Clin. Microbiol. 50, 1663–1672 10.1128/JCM.06540-11 22403426 PMC3347141

[B108] Fleming A. F. Maharajan R. Abraham M. Kulkarni A. G. Bhusnurmath S. R. Okpara R. A. (1986). Antibodies to HTLV-I in Nigerian blood-donors, their relatives and patients with leukaemias, lymphomas and other diseases. Int. J. Cancer 38, 809–813 10.1002/ijc.2910380605 2878890

[B109] Fouchard N. Mahe A. Huerre M. Fraitag S. Valensi F. Macintyre E. (1998). Cutaneous T cell lymphomas: mycosis fungoides, Sezary syndrome and HTLV-I-associated adult T cell leukemia (ATL) in Mali, West Africa: a clinical, pathological and immunovirological study of 14 cases and a review of the African ATL cases. Leukemia 12, 578–585 10.1038/sj.leu.2400956 9557617

[B110] Freeman R. C. Rodriguez G. M. French J. F. (1995). Seroprevalence and risk factors associated with HTLV-I/II infection in injection drug users in northern New Jersey. J. Addict. Dis. 14, 51–66 10.1300/J069v14n03_04 8555279

[B111] Fujiyoshi T. Li H. C. Lou H. Yashiki S. Karino S. Zaninovic V. (1999). Characteristic distribution of HTLV type I and HTLV type II carriers among native ethnic groups in South America. AIDS Res. Hum. Retroviruses 15, 1235–1239 10.1089/088922299310124 10505671

[B112] Furusyo N. Hayashi J. Kakuda K. Sawayama Y. Ariyama I. Eddie R. (1999). Markedly high seroprevalence of hepatitis B virus infection in comparison to hepatitis C virus and human T lymphotropic virus type-1 infections in selected Solomon Islands populations. Am. J. Trop. Med. Hyg. 61, 85–91 10432062 10.4269/ajtmh.1999.61.85

[B113] Gabarre J. Gessain A. Raphael M. Merle-Beral H. Dubourg O. Fourcade C. (1993). Adult T-cell leukemia/lymphoma revealed by a surgically cured cardiac valve lymphomatous involvement in an Iranian woman: clinical, immunopathological and viromolecular studies. Leukemia 7, 1904–1909 8231261

[B114] Galvao-Castro B. Loures L. Rodriques L. G. Sereno A. Ferreira Junior O. C. Franco L. G. (1997). Distribution of human T-lymphotropic virus type I among blood donors: a nationwide Brazilian study. Transfusion 37, 242–243 10.1046/j.1537-2995.1997.37297203532.x 9051104

[B115] Gascoyne R. D. Kim S. M. Oger J. J. Melosky B. L. Dekaban G. A. (1996). HTLV-I associated adult T cell leukemia/lymphoma: report of two cases from an Amerindian population in coastal northwest British Columbia. Leukemia 10, 552–557 8642874

[B116] Gasmi M. Farouqi B. d’Incan M. Desgranges C. (1994). Long terminal repeat sequence analysis of HTLV type I molecular variants identified in four north African patients. AIDS Res. Hum. Retroviruses 10, 1313–1315 10.1089/aid.1994.10.1313 7848687

[B117] Gastaldello R. Hall W. W. Gallego S. (2004). Seroepidemiology of HTLV-I/II in Argentina: an overview. J. Acquir. Immune Defic. Syndr. 35, 301–308 10.1097/00126334-200403010-00012 15076246

[B118] Gerard Y. Lepere J. F. Pradinaud R. Joly F. Lepelletier L. Joubert M. (1995). Clustering and clinical diversity of adult T-cell leukemia/lymphoma associated with HTLV-I in a remote black population of French Guiana. Int. J. Cancer 60, 773–776 10.1002/ijc.2910600607 7896443

[B119] Gessain A. (2011). Human retrovirus HTLV-1: descriptive and molecular epidemiology, origin, evolution, diagnosis and associated diseases. Bull. Soc. Pathol. Exot. 104, 167–180 10.1007/s13149-011-0174-4 21796326

[B120] Gessain A. Caumes E. Feyeux C. d’Agay M. F. Capesius C. Gentilini M. (1992a). The cutaneous form of adult T-cell leukemia/lymphoma in a woman from the Ivory Coast. Clinical, immunovirologic studies and a review of the African adult T-cell leukemia/lymphoma cases. Cancer 69, 1362–1367 10.1002/1097-0142(19920315)69:6<1362::AID-CNCR2820690610>3.0.CO;2-B 1311622

[B121] Gessain A. Gallo R. C. Franchini G. (1992b). Low degree of human T-cell leukemia/lymphoma virus type I genetic drift in vivo as a means of monitoring viral transmission and movement of ancient human populations. J. Virol. 66, 2288–2295 1548762 10.1128/jvi.66.4.2288-2295.1992 PMC289023

[B122] Gessain A. de The G. (1996). What is the situation of human T cell lymphotropic virus type II (HTLV-II) in Africa? Origin and dissemination of genomic subtypes. J. Acquir. Immune Defic. Syndr. Hum. Retrovirol. 13(Suppl. 1), S228–S235 10.1097/00042560-199600001-00022 8797728

[B123] Gessain A. Fretz C. Koulibaly M. Boudret M. L. Bah A. Raphael M. (1993a). Evidence of HTLV-II infection in Guinea, West Africa. J. Acquir. Immune Defic. Syndr. 6, 324–325 8450411

[B124] Gessain A. Herve V. Jeannel D. Garin B. Mathiot C. de-The G. (1993b). HTLV-1 but not HTLV-2 found in pygmies from Central African Republic. J. Acquir. Immune Defic. Syndr. 6, 1373–1374 8254478

[B125] Gessain A. Gout O. Saal F. Daniel M. T. Rio B. Flandrin G. (1990a). Epidemiology and immunovirology of human T-cell leukemia/lymphoma virus type I-associated adult T-cell leukemia and chronic myelopathies as seen in France. Cancer Res. 50, 5692S–5696S 2201441

[B126] Gessain A. Moulonguet I. Flageul B. Perrin P. Capesius C. D’Agay M. F. (1990b). Cutaneous type of adult T cell leukemia/lymphoma in a French West Indian woman. Clonal rearrangement of T-cell receptor beta and gamma genes and monoclonal integration of HTLV-I proviral DNA in the skin infiltrate. J. Am. Acad. Dermatol. 23, 994–1000 10.1016/0190-9622(90)70321-8 2172339

[B127] Gessain A. Saal F. Gout O. Daniel M. T. Flandrin G. de The G. (1990c). High human T-cell lymphotropic virus type I proviral DNA load with polyclonal integration in peripheral blood mononuclear cells of French West Indian, Guianese, and African patients with tropical spastic paraparesis. Blood 75, 428–433 1967218

[B128] Gessain A. Yanagihara R. Franchini G. Garruto R. M. Jenkins C. L. Ajdukiewicz A. B. (1991). Highly divergent molecular variants of human T-lymphotropic virus type I from isolated populations in Papua New Guinea and the Solomon Islands. Proc. Natl. Acad. Sci. U.S.A. 88, 7694–7698 10.1073/pnas.88.17.7694 1881912 PMC52368

[B129] Gharibi L. Marouan S. Zouhair K. Benchekroun M. Benchikhi H. (2011). Fatal erythroderma in a young Moroccan. Med. Trop. (Mars) 71, 189–191 21695884

[B130] Gibbs W. N. Lofters W. S. Campbell M. Hanchard B. LaGrenade L. Clark J. (1984). Adult T-cell leukemia/lymphoma in Jamaica and its relationship to human T-cell leukemia/lymphoma virus type I-associated lymphoproliferative disease. Int. Symp. Princess Takamatsu Cancer Res. Fund 15, 77–90 6100652

[B131] Gioseffi O. N. Nucifora E. Fantl D. Dufour C. Milone J. Di Paolo H. (1995). Adult HTLV-I positive leukemia-lymphoma in Argentina. Sangre (Barc) 40, 421–424 8553178

[B132] Gongora-Biachi R. A. Gonzalez-Martinez P. Castro-Sansores C. Bastarrachea-Ortiz J. (1997). Infection with HTLV virus type I-II in patients with cervico-uterine cancer in the Yucatan peninsula, Mexico. Ginecol. Obstet. Mex. 65, 141–144 9280739

[B133] Gongora-Biachi R. A. Gonzalez-Martinez P. Castro-Sansores C. Vivas-Rosel M. L. Gasque-Lopez F. Garrido-Hadad E. (1996). Lymphotropic viruses type I and II in pregnant women in Yucatan. Rev. Invest. Clin. 48, 383–384 9005516

[B134] Goto K. Sato K. Kurita M. Masuhara N. Iijima Y. Saeki K. (1997). Serologic survey for HTLV-I in Kanagawa Prefecture. Tokai J. Exp. Clin. Med. 22, 7–8 9608625

[B135] Gotuzzo E. Cabrera J. Deza L. Verdonck K. Vandamme A. M. Cairampoma R. (2004). Clinical characteristics of patients in Peru with human T cell lymphotropic virus type 1-associated tropical spastic paraparesis. Clin. Infect. Dis. 39, 939–944 10.1086/423957 15472843

[B136] Goubau P. Carton H. Kazadi K. Muya K. W. Desmyter J. (1990). HTLV seroepidemiology in a central African population with high incidence of tropical spastic paraparesis. Trans. R. Soc. Trop. Med. Hyg. 84, 577–579 10.1016/0035-9203(90)90046-H 2091355

[B137] Goubau P. Desmyter J. Swanson P. Reynders M. Shih J. Surmont I. (1993). Detection of HTLV-I and HTLV-II infection in Africans using type-specific envelope peptides. J. Med. Virol. 39, 28–32 10.1002/jmv.1890390107 8093712

[B138] Gout O. Gessain A. Bolgert F. Saal F. Tournier-Lasserve E. Lasneret J. (1989). Chronic myelopathies associated with human T-lymphotropic virus type I. A clinical, serologic, and immunovirologic study of ten patients in France. Arch. Neurol. 46, 255–260 10.1001/archneur.1989.00520390021009 2919977

[B139] Gracia F. Reeves W. C. Levine P. H. Cuevas M. Castillo L. Chavarria R. (1990). Human T-cell lymphotropic virus type I and neurologic disease in Panama, 1985 and 1986. Arch. Neurol. 47, 634–639 10.1001/archneur.1990.00530060042014 2346389

[B140] Gradilone A. Zani M. Barillari G. Modesti M. Agliano A. M. Maiorano G. (1986). HTLV-I and HIV infection in drug addicts in Italy. Lancet 2, 753–754 10.1016/S0140-6736(86)90275-8 2876222

[B141] Grant W. Bia F. J. Chacko T. M. Jean-Baptiste M. Griffith B. P. (1992). Comparison of enzyme-linked immunosorbent and indirect immunofluorescence assays for the detection of human T-cell lymphotropic virus type-I antibodies in sera from rural Haiti. Diagn. Microbiol. Infect. Dis. 15, 121–124 10.1016/0732-8893(92)90034-Q 1572136

[B142] Group R. S. (1989). Nationwide community-based serological survey of HIV-1 and other human retrovirus infections in a central African country. Rwandan HIV Seroprevalence Study Group. Lancet 1, 941–943 2565428

[B143] Gudo E. S. Abreu C. M. Mussa T. Augusto Ado R. Otsuki K. Chambo E. (2009). Serologic and molecular typing of human T-lymphotropic virus among blood donors in Maputo City, Mozambique. Transfusion 49, 1146–1150 10.1111/j.1537-2995.2009.02100.x 19222818

[B144] Guimaraes de Souza V. Lobato Martins M. de Freitas Carneiro-Proietti A. B. Januario J. N. Ladeira R. V. Silva C. M. (2012). High prevalence of HTLV-1 and 2 viruses in pregnant women in Sao Luis, state of Maranhao, Brazil. Rev. Soc. Bras. Med. Trop. 45, 159–162 10.1590/S0037-86822012000200004 22534984

[B145] Guo H. G. Wong-Stall F. Gallo R. C. (1984). Novel viral sequences related to human T-cell leukemia virus in T cells of a seropositive baboon. Science 223, 1195–1197 10.1126/science.6322297 6322297

[B146] Hale A. Leung T. Sivasubramaniam S. Kenny J. Sutherland S. (1997). Prevalence of antibodies to HTLV in antenatal clinic attenders in south east London. J. Med. Virol. 52, 326–329 10.1002/(SICI)1096-9071(199707)52:3<326::AID-JMV15>3.3.CO;2-Y 9210044

[B147] Haque A. Hossain M. Khan J. K. Kuo Y. H. Lambein F. De Reuck J. (1994). New findings and symptomatic treatment for neurolathyrism, a motor neuron disease occurring in north west Bangladesh. Paraplegia 32, 193–195 10.1038/sc.1994.35 8008424

[B148] Harrington W. J. Jr. Ucar A. Gill P. Snodgrass S. Sheremata W. Cabral L. (1995). Clinical spectrum of HTLV-I in south Florida. J. Acquir. Immune Defic. Syndr. Hum. Retrovirol. 8, 466–473 10.1097/00042560-199504120-00006 7697443

[B149] Hayami M. Komuro A. Nozawa K. Shotake T. Ishikawa K. Yamamoto K. (1984). Prevalence of antibody to adult T-cell leukemia virus-associated antigens (ATLA) in Japanese monkeys and other non-human primates. Int. J. Cancer 33, 179–183 10.1002/ijc.2910330205 6319300

[B150] Hernandez Ramirez P. Rivero Jimenez R. Ballester Santovenia M. Navea Leyva L. Matutes E. Catovsky D. (1991). Very low seroprevalence of HTLV-I/II in Cuba: antibodies in blood donors and in hematological and nonhematological patients. Vox Sang. 61, 277–278 10.1111/j.1423-0410.1991.tb00960.x 1685604

[B151] Hino S. (2011). Establishment of the milk-borne transmission as a key factor for the peculiar endemicity of human T-lymphotropic virus type 1 (HTLV-1): the ATL prevention program Nagasaki. Proc. Jpn. Acad. Ser. B Phys. Biol. Sci. 87, 152–166 10.2183/pjab.87.152 21558754 PMC3149377

[B152] Hino S. Katamine S. Kawase K. Miyamoto T. Doi H. Tsuji Y. (1994). Intervention of maternal transmission of HTLV-1 in Nagasaki, Japan. Leukemia 8 (Suppl. 1), S68–S70 8152307

[B153] Hino S. Sugiyama H. Doi H. Ishimaru T. Yamabe T. Tsuji Y. (1987). Breaking the cycle of HTLV-I transmission via carrier mothers’ milk. Lancet 2, 158–159 10.1016/S0140-6736(87)92358-0 2885619

[B154] Hino S. Yamaguchi K. Katamine S. Sugiyama H. Amagasaki T. Kinoshita K. (1985). Mother-to-child transmission of human T-cell leukemia virus type-I. Jpn. J. Cancer Res. 76, 474–480 2991060

[B155] Hinuma Y. Komoda H. Chosa T. Kondo T. Kohakura M. Takenaka T. (1982). Antibodies to adult T-cell leukemia-virus-associated antigen (ATLA) in sera from patients with ATL and controls in Japan: a nation-wide sero-epidemiologic study. Int. J. Cancer 29, 631–635 10.1002/ijc.2910290606 6980846

[B156] Hlela C. Shepperd S. Khumalo N. P. Taylor G. P. (2009). The prevalence of human T-cell lymphotropic virus type 1 in the general population is unknown. AIDS Rev. 11, 205–214 19940947

[B157] Homma T. Kanki P. J. King N. W. Jr. Hunt R. D. O’Connell M. J. Letvin N. L. (1984). Lymphoma in macaques: association with virus of human T lymphotrophic family. Science 225, 716–718 10.1126/science.6087453 6087453

[B158] Houinato D. Verdier M. Josse R. Zohoun T. Letenneur L. Salamon R. (1996). Seroepidemiological study of retroviruses (HTLV-I/II, HIV-1, HIV-2) in the Department of Atacora, northern Benin. Trop. Med. Int. Health 1, 205–209 10.1111/j.1365-3156.1996.tb00027.x 8665385

[B159] Houston S. Thornton C. Emmanuel J. Latif A. (1994). Human T cell lymphotropic virus type 1 in Zimbabwe. Trans. R. Soc. Trop. Med. Hyg. 88, 170–172 10.1016/0035-9203(94)90281-X 8036662

[B160] Hunsmann G. Bayer H. Schneider J. Schmitz H. Kern P. Dietrich M. (1984). Antibodies to ATLV/HTLV-1 in Africa. Med. Microbiol. Immunol. 173, 167–170 10.1007/BF02123765 6095000

[B161] Hunsmann G. Schneider J. Schmitt J. Yamamoto N. (1983). Detection of serum antibodies to adult T-cell leukemia virus in non-human primates and in people from Africa. Int. J. Cancer 32, 329–332 10.1002/ijc.2910320311 6604034

[B162] Inaba S. Sato H. Okochi K. Fukada K. Takakura F. Tokunaga K. (1989). Prevention of transmission of human T-lymphotropic virus type 1 (HTLV-1) through transfusion, by donor screening with antibody to the virus. One-year experience. Transfusion 29, 7–11 10.1046/j.1537-2995.1989.29189101168.x 2643213

[B163] Ishida T. Hinuma Y. (1986). The origin of Japanese HTLV-I. Nature 322, 504 10.1038/322504b0 3016556

[B164] Ishida T. Yamamoto K. Omoto K. (1988). A seroepidemiological survey of HTLV-1 in the Philippines. Int. J. Epidemiol. 17, 625–628 10.1093/ije/17.3.625 3264823

[B165] Jain P. Gupta S. Prabhash K. Patkar N. Parikh P. M. (2008). Adult T cell leukemia: a typical case from India. Indian J. Cancer 45, 72–73 10.4103/0019-509X.41776 18626153

[B166] Janssen R. S. Kaplan J. E. Khabbaz R. F. Hammond R. Lechtenberg R. Lairmore M. (1991). HTLV-I-associated myelopathy/tropical spastic paraparesis in the United States. Neurology 41, 1355–1357 10.1212/WNL.41.11.1850 1891080

[B167] Jeannel D. Garin B. Kazadi K. Singa L. de The G. (1993). The risk of tropical spastic paraparesis differs according to ethnic group among HTLV-I carriers in Inongo, Zaire. J. Acquir. Immune Defic. Syndr. 6, 840–844 8509984

[B168] Jeannel D. Kourouma K. Fretz C. Zheng Y. M. Ureta V. A. Drame L. (1995). Regional differences in human retroviral infections HIV-1, HIV-2, and HTLV-I/II in rural Guinea (west Africa). J. Acquir. Immune Defic. Syndr. Hum. Retrovirol. 8, 315–318 10.1097/00042560-199503010-00015 7859147

[B169] Jeon H. J. Lee M. J. Jeong Y. K. Lee D. M. Oh Y. K. Kim C. W. (2000). Adult T cell leukemia/lymphoma with lymphopenia in a Korean. J. Korean Med. Sci. 15, 233–239 10803704 10.3346/jkms.2000.15.2.233 PMC3054617

[B170] Jogessar V. B. de Bruyn C. C. Bhigjee A. I. Naicker V. L. Bill P. L. Tait D. (1992). Adult T-cell leukaemia/lymphoma associated with HTLV-I in Natal. S. Afr. Med. J. 81, 528–529 1585227

[B171] Joubert J. van As A. D. Lecatsas G. Bos P. (1991). Human T-lymphotropic virus type I-associated myelopathy. A case report. S. Afr. Med. J. 80, 592–593 1745951

[B172] Kajiyama W. Kashiwagi S. Ikematsu H. Hayashi J. Nomura H. Okochi K. (1986). Intrafamilial transmission of adult T cell leukemia virus. J. Infect. Dis. 154, 851–857 10.1093/infdis/154.5.851 2877031

[B173] Kaplan J. E. Khabbaz R. F. Murphy E. L. Hermansen S. Roberts C. Lal R. (1996). Male-to-female transmission of human T-cell lymphotropic virus types I and II: association with viral load. The Retrovirus Epidemiology Donor Study Group. J. Acquir. Immune Defic. Syndr. Hum. Retrovirol. 12, 193–201 10.1097/00042560-199606010-00014 8680892

[B174] Kawashti M. I. Hindawi S. I. Damanhouri G. A. Rowehy N. G. Bawazeer M. M. Alshawa M. (2005). Serologial screening of human T cell lymphotropic virus I and II (HTLV I/II) in blood banks by immunoblotting and enzyme-immuno assays: to demand or to defeat? Egypt J. Immunol. 12, 137–142 17977218

[B175] Kayembe K. Goubau P. Desmyter J. Vlietinck R. Carton H. (1990). A cluster of HTLV-1 associated tropical spastic paraparesis in Equateur (Zaire): ethnic and familial distribution. J. Neurol. Neurosurg. Psychiatr. 53, 4–10 10.1136/jnnp.53.1.4 2303831 PMC1014089

[B176] Kazanji M. Gessain A. (2003). Human T-cell Lymphotropic Virus types I and II (HTLV-I/II) in French Guiana: clinical and molecular epidemiology. Cad. Saude Publica 19, 1227–1240 10.1590/S0102-311X2003000500002 14666205

[B177] Kchour G. Tarhini M. Kooshyar M. M. El Hajj H. Wattel E. Mahmoudi M. (2009). Phase 2 study of the efficacy and safety of the combination of arsenic trioxide, interferon alpha, and zidovudine in newly diagnosed chronic adult T-cell leukemia/lymphoma (ATL). Blood 113, 6528–6532 10.1182/blood-2009-03-211821 19411628

[B178] Khabbaz R. F. Hartley T. M. Oberle M. W. Rosero-Bixby L. (1990). Seroprevalence of human T-lymphotropic virus type I (HTLV-I) in Costa Rica. AIDS Res. Hum. Retroviruses 6, 959–960 10.1089/aid.1990.6.959 2390337

[B179] Kim J. M. Song Y. G. Oho Y. C. Park H. C. Kwon K. H. Kim E. (1999). Antibodies to human T-cell lymphotropic virus type I (HTLV-I) by particle agglutination (PA) test in Korean blood donors. Yonsei Med. J. 40, 173–177 10333722 10.3349/ymj.1999.40.2.173

[B180] Kimata J. T. Kaneshiro S. A. Kwock D. W. Nakamura S. Kaneshiro M. M. Nakamura J. M. (1989). A seroepidemiologic survey of human T-cell lymphotropic virus type I in two Hawaiian hematologic-oncologic practices. West. J. Med. 150, 300–302 2735036 PMC1026451

[B181] Kinoshita K. Hino S. Amagaski T. Ikeda S. Yamada Y. Suzuyama J. (1984). Demonstration of adult T-cell leukemia virus antigen in milk from three sero-positive mothers. Gann 75, 103–105 6329875

[B182] Kirkland M. A. Frasca J. Bastian I. (1991). Adult T-cell leukaemia lymphoma in an aborigine. Aust. N. Z. J. Med. 21, 739–741 10.1111/j.1445-5994.1991.tb01380.x 1759923

[B183] Kohakura M. Nakada K. Yonahara M. Komoda H. Imai J. Hinuma Y. (1986). Seroepidemiology of the human retrovirus (HTLV/ATLV) in Okinawa where adult T-cell leukemia is highly endemic. Jpn. J. Cancer Res. 77, 21–23 2870044

[B184] Koralnik I. J. Boeri E. Saxinger W. C. Monico A. L. Fullen J. Gessain A. (1994). Phylogenetic associations of human and simian T-cell leukemia/lymphotropic virus type I strains: evidence for interspecies transmission. J. Virol. 68, 2693–2707 7908063 10.1128/jvi.68.4.2693-2707.1994 PMC236747

[B185] Kumar H. Gupta P. K. (2006). Is seroprevalence of HTLV-I/II among blood donors in India relevant? Indian J. Pathol. Microbiol. 49, 532–534 17183844

[B186] Kwon S. Y. Lim A. H. Park J. Y. Han S. H. Cho N. S. (2008). Seroprevalence of human T-lymphotropic virus type 1 and 2 in Korean blood donors. J. Med. Virol. 80, 1864–1867 10.1002/jmv.21260 18712809

[B187] La Grenade L. Hanchard B. Fletcher V. Cranston B. Blattner W. (1990). Infective dermatitis of Jamaican children: a marker for HTLV-I infection. Lancet 336, 1345–1347 10.1016/0140-6736(90)92896-P 1978165

[B188] Laperche S. Worms B. Pillonel J. (2009). Blood safety strategies for human T-cell lymphotropic virus in Europe. Vox Sang. 96, 104–110 10.1111/j.1423-0410.2008.01136.x 19076337

[B189] Larsen O. Andersson S. da Silva Z. Hedegaard K. Sandstrom A. Naucler A. (2000). Prevalences of HTLV-1 infection and associated risk determinants in an urban population in Guinea-Bissau, West Africa. J. Acquir. Immune Defic. Syndr. 25, 157–163 11103046 10.1097/00042560-200010010-00010

[B190] Lavanchy D. Bovet P. Hollanda J. Shamlaye C. F. Burczak J. D. Lee H. (1991). High seroprevalence of HTLV-I in the Seychelles. Lancet 337, 248–249 10.1016/0140-6736(91)92222-N 1670887

[B191] Le Hesran J. Y. Delaporte E. Gaudebout C. Trebuck A. Schrijvers D. Josse R. (1994). Demographic factors associated with HTLV-1 infection in a Gabonese community. Int. J. Epidemiol. 23, 812–817 10.1093/ije/23.4.812 8002196

[B192] Lechat M. F. Shrager D. I. Declercq E. Bertrand F. Blattner W. A. Blumberg B. S. (1997). Decreased survival of HTLV-I carriers in leprosy patients from the Democratic Republic of the Congo: a historical prospective study. J. Acquir. Immune Defic. Syndr. Hum. Retrovirol. 15, 387–390 10.1097/00042560-199708150-00010 9342260

[B193] Lee C. W. Chang M. C. Chang Y. F. Hsieh R. K. Lin J. Chen K. S. (2010). Adult T-cell leukemia/lymphoma in Taiwan: an analysis of 17 patients and review of the literature. Asia Pac. J. Clin. Oncol. 6, 161–164 10.1111/j.1743-7563.2010.01278.x 20887496

[B194] Lee H. H. Weiss S. H. Brown L. S. Mildvan D. Shorty V. Saravolatz L. (1990). Patterns of HIV-1 and HTLV-I/II in intravenous drug abusers from the middle atlantic and central regions of the USA. J. Infect. Dis. 162, 347–352 10.1093/infdis/162.2.347 2373871

[B195] Lee S. Y. Yamaguchi K. Takatsuki K. Kim B. K. Park S. Lee M. (1986). Seroepidemiology of human T-cell leukemia virus type-I in the Republic of Korea. Jpn. J. Cancer Res. 77, 250–254 2871003

[B196] Leon G. Quiros A. M. Lopez J. L. Hung M. Diaz A. M. Goncalves J. (2003). Seropositivity for human T-lymphotropic virus types I and II among donors at the Municipal Blood Bank of Caracas and associated risk factors. Rev. Panam. Salud Publica 13, 117–123 10.1590/S1020-49892003000200012 12744787

[B197] Levine P. H. Reeves W. C. Cuevas M. Arosemena J. R. Jaffe E. S. Saxinger W. C. (1989). Human T-cell leukemia virus-I and hematologic malignancies in Panama. Cancer 63, 2186–2191 10.1002/1097-0142(19890601)63:11<2186::AID-CNCR2820631121>3.0.CO;2-J 2720568

[B198] Li H. C. Biggar R. J. Miley W. J. Maloney E. M. Cranston B. Hanchard B. (2004). Provirus load in breast milk and risk of mother-to-child transmission of human T lymphotropic virus type I. J. Infect. Dis. 190, 1275–1278 10.1086/423941 15346338

[B199] Louis F. J. Sapin C. Huerre M. Desforges S. Labrouasse R. Friquet I. (1990). Prévalence à Wallis des trépanomatoses et des infections à virus HTLV-1 et HB. Médecine d’Océanie 2, 28–32

[B200] Louisiriotchanakul S. Thongput A. Khamboonruang C. Taylor G. P. Kunstadter P. Wasi C. (2000). No evidence of HTLV-I or HTLV-II infection among the Hmong people of northern Thailand or injecting drug users in Bangkok. J. Acquir. Immune Defic. Syndr. 23, 441–442 10.1097/00042560-200004150-00015 10866241

[B201] Lu S. C. Kao C. L. Chin L. T. Chen J. W. Yang C. M. Chang J. H. (2001). Seroprevalence and demographic characteristics of HTLV-I among blood donors in Taiwan: 1996–1999. Int. J. Hematol. 74, 333–337 10.1007/BF02982070 11721972

[B202] Machuca A. Tuset C. Soriano V. Caballero E. Aguilera A. Ortiz de Lejarazu R. (2000). Prevalence of HTLV infection in pregnant women in Spain. Sex. Transm. Infect. 76, 366–370 10.1136/sti.76.5.366 11141853 PMC1744220

[B203] Maeda Y. Furukawa M. Takehara Y. Yoshimura K. Miyamoto K. Matsuura T. (1984). Prevalence of possible adult T-cell leukemia virus-carriers among volunteer blood donors in Japan: a nation-wide study. Int. J. Cancer 33, 717–720 10.1002/ijc.2910330602 6329964

[B204] Maehama T. (2004). Human T cell leukemia virus-1 in pregnancy. Int. J. Gynaecol. Obstet. 87, 247–248 10.1016/j.ijgo.2004.07.024 15548399

[B205] Mahe A. Gessain A. Huerre M. Valensi F. Keita S. Bobin P. (1994). Adult T-cell leukemia associated with HTLV-1 in a HIV-2 seropositive African. Ann. Dermatol. Venereol. 121, 704–709 7793759

[B206] Mahieux R. Chappey C. Georges-Courbot M. C. Dubreuil G. Mauclere P. Georges A. (1998). Simian T-cell lymphotropic virus type 1 from Mandrillus sphinx as a simian counterpart of human T-cell lymphotropic virus type 1 subtype D. J. Virol. 72, 10316–10322 9811783 10.1128/jvi.72.12.10316-10322.1998 PMC110621

[B207] Mahieux R. Gessain A. (2009). The human HTLV-3 and HTLV-4 retroviruses: new members of the HTLV family. Pathol. Biol. 57, 161–166 10.1016/j.patbio.2008.02.015 18456423

[B208] Mahieux R. Gessain A. Truffert A. Vitrac D. Hubert A. Dandelot J. (1994). Seroepidemiology, viral isolation, and molecular characterization of human T cell leukemia/lymphoma virus type I from La Reunion Island, Indian Ocean. AIDS Res. Hum. Retroviruses 10, 745–752 10.1089/aid.1994.10.745 7915530

[B209] Mahieux R. Horal P. Mauclere P. Mercereau-Puijalon O. Guillotte M. Meertens L. (2000). Human T-cell lymphotropic virus type 1 gag indeterminate western blot patterns in Central Africa: relationship to Plasmodium falciparum infection. J. Clin. Microbiol. 38, 4049–4057 11060067 10.1128/jcm.38.11.4049-4057.2000 PMC87540

[B210] Mahieux R. Ibrahim F. Mauclere P. Herve V. Michel P. Tekaia F. (1997). Molecular epidemiology of 58 new African human T-cell leukemia virus type 1 (HTLV-1) strains: identification of a new and distinct HTLV-1 molecular subtype in Central Africa and in Pygmies. J. Virol. 71, 1317–1333 8995656 10.1128/jvi.71.2.1317-1333.1997 PMC191187

[B211] Maloney E. M. Yamano Y. Vanveldhuisen P. C. Sawada T. Kim N. Cranston B. (2006). Natural history of viral markers in children infected with human T lymphotropic virus type I in Jamaica. J. Infect. Dis. 194, 552–560 10.1086/506365 16897651

[B212] Manca N. Ferremi P. De Simone N. Pirali F. Turano A. (1997). Isolation of HTLV-1 from an aggressive form of ATL in a Romanian patient not at risk of infection and with seronegative family members. New Microbiol. 20, 177–185 9258936

[B213] Mansuy J. M. Schlegel L. Villeneuve L. Mengelle C. Magnaval J. F. (1999). Seroprevalence of retroviral infections among pregnant women in Martinique (French West Indies). Am. J. Trop. Med. Hyg. 61, 598–599 10548294 10.4269/ajtmh.1999.61.598

[B214] Marin O. Hasui K. Remondegui C. Sato E. Aye M. M. Takenouchi N. (2002). Adult T-cell leukemia/lymphoma in Jujuy, north-west Argentina. Pathol. Int. 52, 348–357 10.1046/j.1440-1827.2002.01356.x 12100517

[B215] Martin F. Fedina A. Youshya S. Taylor G. P. (2010). A 15-year prospective longitudinal study of disease progression in patients with HTLV-1 associated myelopathy in the UK. J. Neurol. Neurosurg. Psychiatr. 81, 1336–1340 10.1136/jnnp.2009.191239 20660921

[B216] Mauclère P. Afonso P. Meertens L. Plancoulaine S. Calattini S. Froment A. (2011). HTLV-2B strains, similar to those found in several Amerindians tribes, are endemic in Centra African Bakola Pygmies. J. Infect. Dis. 203, 1316–1323 10.1093/infdis/jir031 21459818

[B217] Mauclere P. Le Hesran J. Y. Mahieux R. Salla R. Mfoupouendoun J. Abada E. T. (1997). Demographic, ethnic, and geographic differences between human T cell lymphotropic virus (HTLV) type I-seropositive carriers and persons with HTLV-I gag-indeterminate Western blots in Central Africa. J. Infect. Dis. 176, 505–509 10.1086/514071 9237719

[B218] May J. T. Stent G. Schnagl R. D. (1988). Antibody to human T-cell lymphotropic virus type I in Australian aborigines. Med. J. Aust. 149, 104 2839756 10.5694/j.1326-5377.1988.tb120516.x

[B219] Meertens L. Rigoulet J. Mauclere P. Van Beveren M. Chen G. M. Diop O. (2001). Molecular and phylogenetic analyses of 16 novel simian T cell leukemia virus type 1 from Africa: close relationship of STLV-1 from Allenopithecus nigroviridis to HTLV-1 subtype B strains. Virology 287, 275–285 10.1006/viro.2001.1018 11531406

[B220] Michel P. Develoux M. Talarmin F. Ndiaye P. Ndiaye M. Raphenon G. (1996). Pathologies associated with HTLV-1 virus in Dakar (1992–1995). Med. Trop. (Mars) 56, 249–254 9026591

[B221] Miller M. Achiron A. Shaklai M. Stark P. Maayan S. Hannig H. (1998). Ethnic cluster of HTLV-I infection in Israel among the Mashhadi Jewish population. J. Med. Virol. 56, 269–274 10.1002/(SICI)1096-9071(199811)56:3<269::AID-JMV16>3.0.CO;2-9 9783697

[B222] Miura T. Fukunaga T. Igarashi T. Yamashita M. Ido E. Funahashi S. (1994). Phylogenetic subtypes of human T-lymphotropic virus type I and their relations to the anthropological background. Proc. Natl. Acad. Sci. U.S.A. 91, 1124–1127 10.1073/pnas.91.3.1124 8302841 PMC521466

[B223] Mochizuki M. Watanabe T. Yamaguchi K. Tajima K. Yoshimura K. Nakashima S. (1992). Uveitis associated with human T lymphotropic virus type I: seroepidemiologic, clinical, and virologic studies. J. Infect. Dis. 166, 943–944 10.1093/infdis/166.4.943 1527436

[B224] Mojaat N. Kaabi H. Hmida S. Maamar M. Slama S. Boukef K. (1999). Seroprevalence of HTLV-I/II antibodies in blood donors and different groups at risk in Tunisia. J. Acquir. Immune Defic. Syndr. 22, 314–315 10.1097/00126334-199911010-00018 10770357

[B225] Mowbray J. Mawson S. Chawira A. Skidmore S. Boxall E. Desselberger U. (1989). Epidemiology of human T-cell leukaemia/lymphoma virus type 1 (HTLV-1) infections in a subpopulation of Afro-Caribbean origin in England. J. Med. Virol. 29, 289–295 10.1002/jmv.1890290413 2621455

[B226] Muchinik G. Bouzas M. B. Zapiola I. Decaro J. Garcia L. Gallo D. (1992). HTLV-I and HTLV-II infection in Uruguay. J. Acquir. Immune Defic. Syndr. 5, 743–744 1613675

[B227] Mueller N. (1991). The epidemiology of HTLV-I infection. Cancer Causes Control 2, 37–52 10.1007/BF00052359 1873433

[B228] Murphy E. L. Figueroa J. P. Gibbs W. N. Brathwaite A. Holding-Cobham M. Waters D. (1989). Sexual transmission of human T-lymphotropic virus type I (HTLV-I). Ann. Intern. Med. 111, 555–560 2789009 10.7326/0003-4819-111-7-555

[B229] Murphy E. L. Figueroa J. P. Gibbs W. N. Holding-Cobham M. Cranston B. Malley K. (1991). Human T-lymphotropic virus type I (HTLV-I) seroprevalence in Jamaica. I. Demographic determinants. Am. J. Epidemiol. 133, 1114–1124 2035515 10.1093/oxfordjournals.aje.a115824

[B230] Murphy E. L. Watanabe K. Nass C. C. Ownby H. Williams A. Nemo G. (1999). Evidence among blood donors for a 30-year-old epidemic of human T lymphotropic virus type II infection in the United States. J. Infect. Dis. 180, 1777–1783 10.1086/315139 10558931

[B231] Naman R. Klayme S. Naboulsi M. Mokhbat J. Jradi O. Ramia S. (2002). HTLV-I and HTLV-II infections in volunteer blood donors and high-risk groups in Lebanon. J. Infect. 45, 29–31 10.1053/jinf.2002.1006 12217728

[B232] Nerrienet E. Meertens L. Kfutwah A. Foupouapouognigni Y. Gessain A. (2001). Molecular epidemiology of simian T-lymphotropic virus (STLV) in wild-caught monkeys and apes from Cameroon: a new STLV-1, related to human T-lymphotropic virus subtype F, in a *Cercocebus agilis*. J. Gen. Virol. 82, 2973–2977 11714973 10.1099/0022-1317-82-12-2973

[B233] Nicholson S. R. Efandis T. Dimitrakakis M. Karopoulos A. Lee H. Gust I. D. (1992). HTLV-I infection in selected populations in Australia and the western Pacific region. Med. J. Aust. 156, 878–880 1463486

[B234] Nightingale S. Orton D. Ratcliffe D. Skidmore S. Tosswill J. Desselberger U. (1993). Antenatal survey for the seroprevalence of HTLV-1 infections in the West Midlands, England. Epidemiol. Infect. 110, 379–387 10.1017/S0950268800068321 8472781 PMC2272267

[B235] Oger J. J. Werker D. H. Foti D. J. Dekaban G. A. (1993). HTLV-I associated myelopathy: an endemic disease of Canadian aboriginals of the Northwest Pacific coast? Can. J. Neurol. Sci. 20, 302–306 8313245

[B236] Ohkura S. Yamashita M. Cartier L. Tanabe D. G. Hayami M. Sonoda S. (1999). Identification and phylogenetic characterization of a human T-cell leukaemia virus type I isolate from a native inhabitant (Rapa Nui) of Easter Island. J. Gen. Virol. 80(Pt 8), 1995–2001 10466796 10.1099/0022-1317-80-8-1995

[B237] Oki T. Yoshinaga M. Otsuka H. Miyata K. Sonoda S. Nagata Y. (1992). A sero-epidemiological study on mother-to-child transmission of HTLV-I in southern Kyushu, Japan. Asia Oceania J. Obstet. Gynaecol. 18, 371–377 10.1111/j.1447-0756.1992.tb00333.x 1362873

[B238] Okochi K. Sato H. Hinuma Y. (1984). A retrospective study on transmission of adult T cell leukemia virus by blood transfusion: seroconversion in recipients. Vox Sang. 46, 245–253 10.1111/j.1423-0410.1984.tb00083.x 6328765

[B239] Olaleye D. O. Bernstein L. Sheng Z. Ekweozor C. C. Li X. Y. Sullivan-Halley J. (1994). Type-specific immune response to human T cell lymphotropic virus (HTLV) type I and type II infections in Nigeria. Am. J. Trop. Med. Hyg. 50, 479–486 8166356 10.4269/ajtmh.1994.50.479

[B240] Olaleye D. O. Ekweozor C. C. Sheng Z. Rasheed S. (1995). Evidence of serological cross-reactivities with human immunodeficiency virus types 1 and 2 and human T-lymphotropic virus types I and II in sera of pregnant women in Ibadan, Nigeria. Int. J. Epidemiol. 24, 198–203 10.1093/ije/24.1.198 7797344

[B241] Olbrich Neto J. Meira D. A. (2004). Soroprevalence of HTLV-I/II, HIV, siphylis and toxoplasmosis among pregnant women seen at Botucatu – Sao Paulo – Brazil: risk factors for HTLV-I/II infection. Rev. Soc. Bras. Med. Trop. 37, 28–32 10.1590/S0037-86822004000100008 15042179

[B242] Orland J. R. Engstrom J. Fridey J. Sacher R. A. Smith J. W. Nass C. (2003). Prevalence and clinical features of HTLV neurologic disease in the HTLV outcomes study. Neurology 61, 1588–1594 10.1212/01.WNL.0000096011.92542.DA 14663047

[B243] Osame M. Janssen R. Kubota H. Nishitani H. Igata A. Nagataki S. (1990). Nationwide survey of HTLV-I-associated myelopathy in Japan: association with blood transfusion. Ann. Neurol. 28, 50–56 10.1002/ana.410280110 2375633

[B244] Ouattara S. A. Gody M. de-The G. (1989). Prevalence of HTLV-I compared to HIV-1 and HIV-2 antibodies in different groups in the Ivory Coast (West Africa). J. Acquir. Immune Defic. Syndr. 2, 481–485 2552069

[B245] Padua E. Rodes B. Perez-Pinar T. Silva A. F. Jimenez V. Ferreira F. (2011). Molecular characterization of human T cell leukemia virus type 1 subtypes in a group of infected individuals diagnosed in Portugal and Spain. AIDS Res. Hum. Retroviruses 27, 317–322 10.1089/aid.2010.0195 20950257

[B246] Park J. H. Lee B. I. Chun S. I. Osame M. (1991). A case of HTLV-I associated myelopathy(HAM) in Korea. Yonsei Med. J. 32, 190–194 1949923 10.3349/ymj.1991.32.2.190

[B247] Paun L. Ispas O. Del Mistro A. Chieco-Bianchi L. (1994). HTLV-I in Romania. Eur. J. Haematol. 52, 117–118 10.1111/j.1600-0609.1994.tb01297.x 8119382

[B248] Payne L. J. Tosswill J. H. Taylor G. P. Zuckerman M. Simms I. (2004). In the shadow of HIV-HTLV infection in England and Wales, 1987–2001. Commun. Dis. Public Health 7, 200–206 15481213

[B249] Pepin J. Labbe A. C. Mamadou-Yaya F. Mbelesso P. Mbadingai S. Deslandes S. (2010). Iatrogenic transmission of human T cell lymphotropic virus type 1 and hepatitis C virus through parenteral treatment and chemoprophylaxis of sleeping sickness in colonial Equatorial Africa. Clin. Infect. Dis. 51, 777–784 10.1086/656233 20735238

[B250] Peters A. A. Coulthart M. B. Oger J. J. Waters D. J. Crandall K. A. Baumgartner A. A. (2000). HTLV type I/II in British Columbia Amerindians: a seroprevalence study and sequence characterization of an HTLV type IIa isolate. AIDS Res. Hum. Retroviruses 16, 883–892 10.1089/08892220050042828 10875614

[B251] Picard F. J. Coulthart M. B. Oger J. King E. E. Kim S. Arp J. (1995). Human T-lymphotropic virus type 1 in coastal natives of British Columbia: phylogenetic affinities and possible origins. J. Virol. 69, 7248–7256 7474147 10.1128/jvi.69.11.7248-7256.1995 PMC189647

[B252] Plancoulaine S. Buigues R. P. Murphy E. L. van Beveren M. Pouliquen J. F. Joubert M. (1998). Demographic and familial characteristics of HTLV-1 infection among an isolated, highly endemic population of African origin in French Guiana. Int. J. Cancer 76, 331–336 10.1002/(SICI)1097-0215(19980504)76:3<331::AID-IJC8>3.3.CO;2-0 9579568

[B253] Plumelle Y. Pascaline N. Nguyen D. Panelatti G. Jouannelle A. Jouault H. (1993). Adult T-cell leukemia-lymphoma: a clinico-pathologic study of twenty-six patients from Martinique. Hematol. Pathol. 7, 251–262 8113152

[B254] Poiesz B. J. Papsidero L. D. Ehrlich G. Sherman M. Dube S. Poiesz M. (2001). Prevalence of HTLV-I-associated T-cell lymphoma. Am. J. Hematol. 66, 32–38 10.1002/1096-8652(200101)66:1<32::AID-AJH1004>3.0.CO;2-3 11426489

[B255] Poiesz B. J. Ruscetti F. W. Gazdar A. F. Bunn P. A. Minna J. D. Gallo R. C. (1980). Detection and isolation of type C retrovirus particles from fresh and cultured lymphocytes of a patient with cutaneous T-cell lymphoma. Proc. Natl. Acad. Sci. U.S.A. 77, 7415–7419 10.1073/pnas.77.12.7415 6261256 PMC350514

[B256] Polizzotto M. N. Wood E. M. Ingham H. Keller A. J. (2008). Reducing the risk of transfusion-transmissible viral infection through blood donor selection: the Australian experience 2000 through 2006. Transfusion 48, 55–63 17894794 10.1111/j.1537-2995.2007.01482.x

[B257] Poljak M. Bednarik J. Rednak K. Seme K. Kristancic L. Celan-Lucu B. (1998). Seroprevalence of human T cell leukaemia/lymphoma virus type I (HTLV-I) in pregnant women, patients attending venereological outpatient services and intravenous drug users from Slovenia. Folia Biol. (Praha) 44, 23–25 10730871

[B258] Pombo De Oliveira M. S. (1996). HTLV-1 infection and adultT-cell leukemia in Brazil: an overview. Sao. Paulo Med. J. 114, 1177–1185 9181750 10.1590/s1516-31801996000300007

[B259] Porter K. R. Anthony R. L. Solihin A. Hayes C. G. (1995). Mapping of a human T-lymphotropic virus type I gag protein epitope that cross-reacts with anti-Plasmodium falciparum antibodies. J. Med. Virol. 45, 469–474 10.1002/jmv.1890450419 7545215

[B260] Pouliquen J. F. Hardy L. Lavergne A. Kafiludine E. Kazanji M. (2004). High seroprevalence of human T-cell lymphotropic virus type 1 in blood donors in Guyana and molecular and phylogenetic analysis of new strains in the Guyana shelf (Guyana, Suriname, and French Guiana). J. Clin. Microbiol. 42, 2020–2026 10.1128/JCM.42.5.2020-2026.2004 15131164 PMC404635

[B261] Power C. Weinshenker B. G. Dekaban G. A. Ebers G. C. Francis G. S. Rice G. P. (1989). HTLV-1 associated myelopathy in Canada. Can. J. Neurol. Sci. 16, 330–335 2766126 10.1017/s0317167100029176

[B262] Proietti F. A. Carneiro-Proietti A. B. Catalan-Soares B. C. Murphy E. L. (2005). Global epidemiology of HTLV-I infection and associated diseases. Oncogene 24, 6058–6068 10.1038/sj.onc.1208968 16155612

[B263] Qiu X. Hodges S. Lukaszewska T. Hino S. Arai H. Yamaguchi J. (2008). Evaluation of a new, fully automated immunoassay for detection of HTLV-I and HTLV-II antibodies. J. Med. Virol. 80, 484–493 10.1002/jmv.21083 18205214

[B264] Quispe N. C. Feria E. B. Santos-Fortuna Ede L. Caterino-de-Araujo A. (2009). Confirming the presence of HTLV-1 infection and the absence of HTLV-2 in blood donors from Arequipa, Peru. Rev. Inst. Med. Trop. Sao Paulo 51, 25–29 10.1590/S0036-46652009000100005 19229387

[B265] Rafatpanah H. Hedayati-Moghaddam M. R. Fathimoghadam F. Bidkhori H. R. Shamsian S. K. Ahmadi S. (2011). High prevalence of HTLV-I infection in Mashhad, Northeast Iran: a population-based seroepidemiology survey. J. Clin. Virol. 52, 172–176 10.1016/j.jcv.2011.07.004 21840754

[B266] Rajabalendaran N. Burns R. Mollison L. C. Blessing W. Kirubakaran M. G. Lindschau P. (1993). Tropical spastic paraparesis in an aborigine. Med. J. Aust. 159, 28–29 8316109 10.5694/j.1326-5377.1993.tb137700.x

[B267] Ramalingam S. Kannangai R. Prakash K. J. Ajithkumar K. Jacob M. George R. (2001). A pilot study of HTLV-I infection in high-risk individuals & their family members from India. Indian J. Med. Res. 113, 201–209 11816953

[B268] Re M. C. Tommaseo M. Furlini G. La Placa M. (1989). High prevalence of serum antibody against human T cell leukemia virus type I (HTLV-I) among the Bismam Asmat population (Indonesian New Guinea). AIDS Res. Hum. Retroviruses 5, 551–554 10.1089/aid.1989.5.551 2590558

[B269] Reddy D. Okochi K. Woodfield D. G. Judd W. (1987). Absence of HTLV I from New Zealand. J. Med. Virol. 22, 375–378 10.1002/jmv.1890220410 3040900

[B270] Reeves W. C. Levine P. H. Cuevas M. Quiroz E. Maloney E. Saxinger W. C. (1990). Seroepidemiology of human T cell lymphotropic virus in the Republic of Panama. Am. J. Trop. Med. Hyg. 42, 374–379 1970459 10.4269/ajtmh.1990.42.374

[B271] Riedel D. A. Evans A. S. Saxinger C. Blattner W. A. (1989). A historical study of human T lymphotropic virus type I transmission in Barbados. J. Infect. Dis. 159, 603–609 10.1093/infdis/159.4.603 2538516

[B272] Rio B. Louvet C. Gessain A. Dormont D. Gisselbrecht C. Martoia R. (1990). Adult T-cell leukemia and non-malignant adenopathies associated with HTLV I virus. Apropos of 17 patients born in the Caribbean region and Africa. Presse Med. 19, 746–751 2140159

[B273] Rodgers-Johnson P. Morgan O. S. Mora C. Sarin P. Ceroni M. Piccardo P. (1988). The role of HTLV-I in tropical spastic paraparesis in Jamaica. Ann. Neurol. 23(Suppl.), S121–S126 10.1002/ana.410230729 2894801

[B274] Roman G. C. Schoenberg B. S. Madden D. L. Sever J. L. Hugon J. Ludolph A. (1987). Human T-lymphotropic virus type I antibodies in the serum of patients with tropical spastic paraparesis in the Seychelles. Arch. Neurol. 44, 605–607 10.1001/archneur.1987.00520160074018 2883962

[B275] Roucoux D. F. Wang B. Smith D. Nass C. C. Smith J. Hutching S. T. (2005). A prospective study of sexual transmission of human T lymphotropic virus (HTLV)-I and HTLV-II. J. Infect. Dis. 191, 1490–1497 10.1086/429410 15809908

[B276] Rouet F. Foucher C. Rabier M. Gawronski I. Taverne D. Chancerel B. (1999a). Human T-lymphotropic virus type I among blood donors from Guadeloupe: donation, demographic, and biologic characteristics. Transfusion 39, 639–644 10.1046/j.1537-2995.1999.39060639.x 10378845

[B277] Rouet F. Rabier R. Foucher C. Chancerel B. Agis F. Strobel M. (1999b). Geographical clustering of human T-cell lymphotropic virus type I in Guadeloupe, an endemic Caribbean area. Int. J. Cancer 81, 330–334 10.1002/(SICI)1097-0215(19990505)81:3<330::AID-IJC3>3.0.CO;2-2 10209944

[B278] Safai B. Huang J. L. Boeri E. Farid R. Raafat J. Schutzer P. (1996). Prevalence of HTLV type I infection in Iran: a serological and genetic study. AIDS Res. Hum. Retroviruses 12, 1185–1190 10.1089/aid.1996.12.1185 8844023

[B279] Saito M. Moritoyo T. Park J. H. Lee B. I. Kim J. S. Fujisawa J. (1993). Nucleotide sequence analysis of HTLV-I isolate from a Korean patient with HAM/TSP. Yonsei Med. J. 34, 321–327 8128736 10.3349/ymj.1993.34.4.321

[B280] Salemi M. Van Dooren S. Audenaert E. Delaporte E. Goubau P. Desmyter J. (1998). Two new human T-lymphotropic virus type I phylogenetic subtypes in seroindeterminates, a Mbuti pygmy and a Gabonese, have closest relatives among African STLV-I strains. Virology 246, 277–287 10.1006/viro.1998.9215 9657946

[B281] Sanchez-Palacios C. Gotuzzo E. Vandamme A. M. Maldonado Y. (2003). Seroprevalence and risk factors for human T-cell lymphotropic virus (HTLV-I) infection among ethnically and geographically diverse Peruvian women. Int. J. Infect. Dis. 7, 132–137 10.1016/S1201-9712(03)90009-9 12839715

[B282] Sanders R. C. Wai’in P. M. Alexander S. S. Levin A. G. Blattner W. A. Alpers M. P. (1993). The prevalence of antibodies to human T-lymphotropic virus type I in different population groups in Papua New Guinea. Arch. Virol. 130, 327–334 10.1007/BF01309664 8517792

[B283] Sarkodie F. Adarkwa M. Adu-Sarkodie Y. Candotti D. Acheampong J. W. Allain J. P. (2001). Screening for viral markers in volunteer and replacement blood donors in West Africa. Vox Sang. 80, 142–147 10.1046/j.1423-0410.2001.00023.x 11840974

[B284] Satake M. Yamaguchi K. Tadokoro K. (2012). Current prevalence of HTLV-1 in Japan as determined by screening of blood donors. J. Med. Virol. 84, 327–335 10.1002/jmv.23181 22170555

[B285] Saxinger W. Blattner W. A. Levine P. H. Clark J. Biggar R. Hoh M. (1984). Human T-cell leukemia virus (HTLV-I) antibodies in Africa. Science 225, 1473–1476 10.1126/science.6089348 6089348

[B286] Schill P. H. Bruneau B. Le Page B. Humeau O. Grimault C. Tampreau V. (1989). Seroprevalence of anti-HIV antibodies in a rural Haitian population. Bull. Soc. Pathol. Exot. Filiales 82, 308–315 2766441

[B287] Schreiber G. B. Murphy E. L. Horton J. A. Wright D. J. Garfein R. Chien H. C. (1997). Risk factors for human T-cell lymphotropic virus types I and II (HTLV-I and -II) in blood donors: the retrovirus epidemiology donor study. NHLBI retrovirus epidemiology donor study. J. Acquir. Immune Defic. Syndr. Hum. Retrovirol. 14, 263–271 10.1097/00042560-199703010-00011 9117460

[B288] Schrijvers D. Delaporte E. Peeters M. Dupont A. Meheus A. (1991). Seroprevalence of retroviral infection in women with different fertility statuses in Gabon, western equatorial Africa. J. Acquir. Immune Defic. Syndr. 4, 468–470 2016684

[B289] Segurado A. Granade T. Parekh B. Nunez C. A. Meza R. Amador L. (1997). Presence of HTLV-I and HTLV-II infection in Honduras. J. Acquir. Immune Defic. Syndr. Hum. Retrovirol. 16, 308 10.1097/00042560-199712010-00014 9402080

[B290] Sene-Diouf F. Ndiaye M. Diop A. G. Thiam A. Ndao A. K. Diagne M. (2000). Epidemiological, clinical and progressive aspects of neurological manifestations associated with retroviral infections: eleven year retrospective study. Dakar Med. 45, 162–166 15779176

[B291] Seniuta N. B. Iakovleva L. S. Stepina V. N. Buachidze L. N. Gurova E. P. Kologrivova Z. A. (1990). Screening of sera from the adult populations of some USSR regions for antibodies to the human T-cell leukemia virus type I (HTLV-I). Vopr. Virusol. 35, 309–312 2256315

[B292] Senyuta N. Syrtsev A. Yamashita M. Stepina V. Susova O. Scherbak L. (1998). Sero-epidemiologic and phylogenetic studies of HTLV-I infection in 2 countries of the Caspian Sea region. Int. J. Cancer 77, 488–493 10.1002/(SICI)1097-0215(19980812)77:4<488::AID-IJC2>3.3.CO;2-G 9679746

[B293] Sertoz R. Turhan A. Bozkurt H. Samlioglu P. Degirmenci A. Aydinok Y. (2010). Investigation of anti-HTLV I/II seroprevalence in healthy blood donors in Izmir region, Turkey. Mikrobiyol. Bul. 44, 579–584 21063970

[B294] Seyfert S. Faulstich A. Marx P. (2004). What determines the CSF concentrations of albumin and plasma-derived IgG? J. Neurol. Sci. 219, 31–33 10.1016/j.jns.2003.12.002 15050434

[B295] Shtalrid M. Shvidel L. Korenfeld R. Duek A. Landau Z. Berrebi A. (2005). HTLV-1 associated adult T-cell leukemia/lymphoma in Israel: report of two patients of Romanian origin. Haematologica 90, ECR13 15753054

[B296] Sidi Y. Meytes D. Shohat B. Fenig E. Weisbort Y. Lee H. (1990). Adult T-cell lymphoma in Israeli patients of Iranian origin. Cancer 65, 590–593 10.1002/1097-0142(19900201)65:3<590::AID-CNCR2820650334>3.0.CO;2-2 2297649

[B297] Silva Cabrera E. Perez Guevara M. T. Lubian Caballero A. L. de la Fuente Arzola J. L. Navea Leyva L. Cruz Sui O. (1997). Search for antibodies against human T-cell lymphotropic virus type I (HTLV-I) in blood donors and risk groups. Rev. Cubana. Med. Trop. 49, 24–27 9685956

[B298] Singhal B. S. Lalkaka J. A. Sonoda S. Hashimoto K. Nomoto M. Kubota R. (1993). Human T-lymphotropic virus type I infections in western India. AIDS 7, 138–139 10.1097/00002030-199301000-00029 8442910

[B299] Stienlauf S. Yahalom V. Schwartz E. Shinar E. Segal G. Sidi Y. (2009). Epidemiology of human T-cell lymphotropic virus type 1 infection in blood donors, Israel. Emerging Infect. Dis. 15, 1116–1118 10.3201/eid1507.080796 19624934 PMC2744246

[B300] Stuver S. O. Tachibana N. Okayama A. Shioiri S. Tsunetoshi Y. Tsuda K. (1993). Heterosexual transmission of human T cell leukemia/lymphoma virus type I among married couples in southwestern Japan: an initial report from the Miyazaki Cohort Study. J. Infect. Dis. 167, 57–65 10.1093/infdis/167.1.57 8418183

[B301] Tajima K. (1990). The 4th nation-wide study of adult T-cell leukemia/lymphoma (ATL) in Japan: estimates of risk of ATL and its geographical and clinical features. The T- and B-cell Malignancy Study Group. Int. J. Cancer 45, 237–243 10.1002/ijc.2910450206 2303290

[B302] Tajima K. Kamura S. Ito S. Ito M. Nagatomo M. Kinoshita K. (1987). Epidemiological features of HTLV-I carriers and incidence of ATL in an ATL-endemic island: a report of the community-based co-operative study in Tsushima, Japan. Int. J. Cancer 40, 741–746 10.1002/ijc.2910400605 2891624

[B303] Takahashi K. Takezaki T. Oki T. Kawakami K. Yashiki S. Fujiyoshi T. (1991). Inhibitory effect of maternal antibody on mother-to-child transmission of human T-lymphotropic virus type I. The Mother-to-Child Transmission Study Group. Int. J. Cancer 49, 673–677 10.1002/ijc.2910490508 1937953

[B304] Takao S. Ishida T. Bhatia K. K. Saha N. Soemantri A. Kayame O. W. (2000). Seroprevalence of human T-lymphotropic virus type 1 in Papua New Guinea and Irian Jaya measured using different western blot criteria. J. Clin. Virol. 16, 129–133 10.1016/S1386-6532(99)00087-6 10720817

[B305] Takezaki T. Tajima K. Komoda H. Imai J. (1995). Incidence of human T lymphotropic virus type I seroconversion after age 40 among Japanese residents in an area where the virus is endemic. J. Infect. Dis. 171, 559–565 10.1093/infdis/171.3.559 7876601

[B306] Tanggo Y. Gultom S. P. Simanjuntak T. Sibuea W. H. Matsuzaki H. Yamaguchi K. (2000). Human T lymphotropic virus I in Indonesia. Very low seroprevalence in the Jakarta area: antibodies in healthy blood donors and in various nonhematological diseases. Intervirology 43, 77–79 10.1159/000025027 10971124

[B307] Taylor G. P. Bodeus M. Courtois F. Pauli G. Del Mistro A. Machuca A. (2005). The seroepidemiology of human T-lymphotropic viruses: types I and II in Europe: a prospective study of pregnant women. J. Acquir. Immune Defic. Syndr. 38, 104–109 10.1097/00126334-200501010-00018 15608533

[B308] Taylor M. B. Parker S. P. Crewe-Brown H. H. McIntyre J. Cubitt W. D. (1996). Seroepidemiology of HTLV-I in relation to that of HIV-1 in the Gauteng region, South Africa, using dried blood spots on filter papers. Epidemiol. Infect. 117, 343–348 10.1017/S0950268800001527 8870632 PMC2271693

[B309] Temple J. J. Brammer M. G. Andes W. A. Covington S. Rangan S. (1986). Adult T-cell leukemia-lymphoma. Unusual features of two patients from a low-incidence area. Cancer 58, 694–698 3015369 10.1002/1097-0142(19860801)58:3<694::aid-cncr2820580316>3.0.co;2-w

[B310] Thyss A. Michiels J. F. Ayela P. Lagrange M. Hoffman P. Schneider M. (1990). Leukemia/lymphoma T syndrome associated with HTLV 1 in a patient of Moroccan origin. Presse Med. 19, 135 2137595

[B311] Toro C. Rodes B. Aguilera A. Caballero E. Benito R. Tuset C. (2002). Clinical impact of HTLV-1 infection in Spain: implications for public health and mandatory screening. J. Acquir. Immune Defic. Syndr. 30, 366–368 10.1097/00042560-200207010-00016 12131577

[B312] Tortevoye P. Tuppin P. Carles G. Peneau C. Gessain A. (2005). Comparative trends of seroprevalence and seroincidence rates of human T cell lymphotropic virus type I and human immunodeficiency virus 1 in pregnant women of various ethnic groups sharing the same environment in French Guiana. Am. J. Trop. Med. Hyg. 73, 560–565 16172481

[B313] Tortevoye P. Tuppin P. Peneau C. Carles G. Gessain A. (2000). Decrease of human T-cell lymphotropic virus type I prevalence and low incidence among pregnant women from a high endemic ethnic group in French Guiana. Int. J. Cancer 87, 534–538 10.1002/1097-0215(20000815)87:4<534::AID-IJC12>3.3.CO;2-D 10918194

[B314] Trejo-Avila L. M. Fuentes-Pensamiento R. Scorro Flores-Castaneda M. Diaz-Mendoza M. L. Zapata-Benavides P. Tamez-Guerra R. S. (1996). Seroprevalence of HTLV-I/II in blood donors in Monterrey, Mexico. Arch. Med. Res. 27, 97–99 8867376

[B315] Trenchi A. Gastaldello R. Balangero M. Irizar M. Cudola A. Gallego S. (2007). Retrospective study of the prevalence of human T-cell lymphotropic virus-type 1/2, HIV, and HBV in pregnant women in Argentina. J. Med. Virol. 79, 1974–1978 10.1002/jmv.21027 17935192

[B316] Trevino A. Aguilera A. Caballero E. Benito R. Parra P. Eiros J. M. (2012). Trends in the prevalence and distribution of HTLV-1 and HTLV-2 infections in Spain. Virol. J. 9, 71 10.1186/1743-422X-9-71 22444832 PMC3337814

[B317] Trujillo J. M. Concha M. Munoz A. Bergonzoli G. Mora C. Borrero I. (1992). Seroprevalence and cofactors of HTLV-I infection in Tumaco, Colombia. AIDS Res. Hum. Retroviruses 8, 651–657 10.1089/aid.1992.8.651 1515216

[B318] Tseliou P. M. Spanakis N. Spiliotakara A. Markogiannakis A. Legakis N. J. Tsakris A. (2006). Prevalence of infection by HTLV-I/II among pregnant women and high-risk groups in the Peloponnese peninsula, Greece. Int. J. STD AIDS 17, 543–546 10.1258/095646206778145541 16925902

[B319] Tseliou P. M. Spiliotakara A. Politis C. Spanakis N. Legakis N. J. Tsakris A. (2004). Prevalence of human T-cell lymphotropic virus-I/II-indeterminate reactivities in a Greek blood bank population. Transfus Med 14, 253–254 10.1111/j.0958-7578.2004.00509.x 15180821

[B320] Tuppin P. Makuwa M. Guerma T. Bazabana M. M. Loukaka J. C. Jeannel D. (1996). Low HTLV-I/II seroprevalence in pregnant women in Congo and a geographic cluster of an HTLV-like indeterminate western blot pattern. J. Acquir. Immune Defic. Syndr. Hum. Retrovirol. 11, 105–107 10.1097/00042560-199601010-00014 8528728

[B321] Tuset C. Gutierrez M. Carbonell C. Tuset T. Soriano V. (1997). Human T-cell lymphotropic virus infection in pregnant women in Spain. Eur. J. Clin. Microbiol. Infect. Dis. 16, 771–773 10.1007/BF01709264 9405953

[B322] Ueda K. Kusuhara K. Tokugawa K. Miyazaki C. Yoshida C. Tokumura K. (1989). Cohort effect on HTLV-I seroprevalence in southern Japan. Lancet 2, 979 10.1016/S0140-6736(89)90986-0 2571887

[B323] Umemoto M. Take H. Kusuhara K. Kuraya K. (1994). Marriage patterns among HTLV-I seropositive women in Japan. Cancer Lett. 81, 237–240 10.1016/0304-3835(94)90208-9 8012942

[B324] Ureta-Vidal A. Angelin-Duclos C. Tortevoye P. Murphy E. Lepere J. F. Buigues R. P. (1999). Mother-to-child transmission of human T-cell-leukemia/lymphoma virus type I: implication of high antiviral antibody titer and high proviral load in carrier mothers. Int. J. Cancer 82, 832–836 10.1002/(SICI)1097-0215(19990909)82:6<832::AID-IJC11>3.0.CO;2-P 10446450

[B325] Vallejo A. Dubon J. M. Garcia-Saiz A. (1996). Presence of human T-cell lymphotropic virus types I and II infections in Honduras. J. Acquir. Immune Defic. Syndr. Hum. Retrovirol. 12, 529–530 10.1097/00042560-199608150-00019 8757437

[B326] van der Ryst E. Joubert G. Smith M. S. Terblanche M. Mollentze F. Pretorius A. M. (1996). HTLV-I infection in the Free State region of South Africa: a sero-epidemiologic study. Cent. Afr. J. Med. 42, 65–68 8653770

[B327] van Doornum G. J. Hooykaas C. Huisman J. G. van der Linden M. M. Coutinho R. A. (1990). Prevalence of human T-cell leukemia virus antibody among heterosexuals living in Amsterdam, The Netherlands. J. Med. Virol. 32, 183–188 10.1002/jmv.1890320309 2280259

[B328] Vandamme A. M. Salemi M. Desmyter J. (1998). The simian origins of the pathogenic human T-cell lymphotropic virus type I. Trends Microbiol. 6, 477–483 10.1016/S0966-842X(98)01406-1 10036726

[B329] Vasquez P. Sanchez G. Volante C. Vera L. Ramirez E. Soto G. (1991). Human T-lymphotropic virus type I: new risk for Chilean population. Blood 78, 850–851 1859897

[B330] Veelken H. Kohler G. Schneider J. Dierbach H. Mertelsmann R. Schaefer H. E. (1996). HTLV-I-associated adult T cell leukemia/lymphoma in two patients from Bucharest, Romania. Leukemia 10, 1366–1369 8709646

[B331] Verdier M. Bonis J. Denis F. (1994). “The Prevalence and Incidence of HTLVs in Africa,” in AIDS in Africa, eds Essex M. Mboup S. Kanki P. Kalengayi M. (New York: Raven Press, Ltd.), 173–192

[B332] Verdier M. Denis F. Sangare A. Barin F. Gershy-Damet G. Rey J. L. (1989). Prevalence of antibody to human T cell leukemia virus type 1 (HTLV-1) in populations of Ivory Coast, West Africa. J. Infect. Dis. 160, 363–370 10.1093/infdis/160.3.363 2547879

[B333] Verdonck K. Gonzalez E. Van Dooren S. Vandamme A. M. Vanham G. Gotuzzo E. (2007). Human T-lymphotropic virus 1: recent knowledge about an ancient infection. Lancet. Infect. Dis. 7, 266–281 10.1016/S1473-3099(07)70081-6 17376384

[B334] Vernant J. C. Maurs L. Gessain A. Barin F. Gout O. Delaporte J. M. (1987). Endemic tropical spastic paraparesis associated with human T-lymphotropic virus type I: a clinical and seroepidemiological study of 25 cases. Ann. Neurol. 21, 123–130 10.1002/ana.410210204 3030190

[B335] Voevodin A. al-Mufti S. Farah S. Khan R. Miura T. (1995). Molecular characterization of human T-lymphotropic virus, type 1 (HTLV-1) found in Kuwait: close similarity with HTLV-1 isolates originating from Mashhad, Iran. AIDS Res. Hum. Retroviruses 11, 1255–1259 10.1089/aid.1995.11.1255 8573383

[B336] Vrielink H. Reesink H. W. (2004). HTLV-I/II prevalence in different geographic locations. Transfus. Med. Rev. 18, 46–57 10.1016/j.tmrv.2003.10.004 14689377

[B337] Wang H. R. Yan Y. S. Zhang Q. W. Zheng J. Liu J. M. Feng Y. Y. (2004). Sero-epidemiological study on the human T-cell leukaemia virus type I/II infection in the east coastal areas of Fujian province. Zhonghua Liu Xing Bing Xue Za Zhi 25, 428–430 15231172

[B338] Wang Y. Li X. Song A. Zhang C. Chen Y. Chen C. (2005). Prevalence and partial sequence analysis of human T cell lymphotropic virus type I in China. J. Med. Virol. 76, 613–618 10.1002/jmv.20405 15977241

[B339] Watanabe T. (2011). Current status of HTLV-1 infection. Int. J. Hematol. 94, 430–434 10.1007/s12185-011-0934-4 21969187

[B340] Watanabe T. Seiki M. Tsujimoto H. Miyoshi I. Hayami M. Yoshida M. (1985). Sequence homology of the simian retrovirus genome with human T-cell leukemia virus type I. Virology 144, 59–65 10.1016/0042-6822(85)90304-6 2998047

[B341] Whyte G. S. (1997). Is screening of Australian blood donors for HTLV-I necessary? Med. J. Aust. 166, 478–481 9152342 10.5694/j.1326-5377.1997.tb123220.x

[B342] Wiktor S. Z. Pate E. J. Murphy E. L. Palker T. J. Champegnie E. Ramlal A. (1993). Mother-to-child transmission of human T-cell lymphotropic virus type I (HTLV-I) in Jamaica: association with antibodies to envelope glycoprotein (gp46) epitopes. J. Acquir. Immune Defic. Syndr. 6, 1162–1167 7692038

[B343] Wiktor S. Z. Piot P. Mann J. M. Nzilambi N. Francis H. Vercauteren G. (1990). Human T cell lymphotropic virus type I (HTLV-I) among female prostitutes in Kinshasa, Zaire. J. Infect. Dis. 161, 1073–1077 10.1093/infdis/161.6.1073 2345292

[B344] Williams A. E. Fang C. T. Slamon D. J. Poiesz B. J. Sandler S. G. Darr W. F. II (1988). Seroprevalence and epidemiological correlates of HTLV-I infection in U.S. blood donors. Science 240, 643–646 10.1126/science.2896386 2896386

[B345] Williams C. K. Alexander S. S. Bodner A. Levine A. Saxinger C. Gallo R. C. (1993). Frequency of adult T-cell leukaemia/lymphoma and HTLV-I in Ibadan, Nigeria. Br. J. Cancer 67, 783–786 10.1038/bjc.1993.142 8471436 PMC1968344

[B346] Williams C. K. Dada A. Blattner W. A. (1994). Some epidemiological features of the human T-cell lymphotropic virus type I (HTLV-I) and ATL in Nigerians. Leukemia 8 (Suppl. 1), S77–S82 8152310

[B347] Wolfe N. D. Heneine W. Carr J. K. Garcia A. D. Shanmugam V. Tamoufe U. (2005). Emergence of unique primate T-lymphotropic viruses among central African bushmeat hunters. Proc. Natl. Acad. Sci. U.S.A. 102, 7994–7999 10.1073/pnas.0501734102 15911757 PMC1142377

[B348] Yamaguchi K. Inaoka T. Ohtsuka R. Akimichi T. Hongo T. Kawabe T. (1993). HTLV-I, HIV-I, and hepatitis B and C viruses in Western Province, Papua New Guinea: a serological survey. Jpn. J. Cancer Res. 84, 715–719 10.1111/j.1349-7006.1993.tb02828.x 7690354 PMC5919200

[B349] Yanagihara E. T. Blaisdell R. K. Hayashi T. Lukes R. J. (1989). Malignant lymphoma in Hawaii-Japanese: a retrospective morphologic survey. Hematol. Oncol. 7, 219–232 10.1002/hon.2900070210 2707745

[B350] Yanagihara R. (1994). Geographic-specific genotypes or topotypes of human T-cell lymphotropic virus type I as markers for early and recent migrations of human populations. Adv. Virus Res. 43, 147–186 10.1016/S0065-3527(08)60048-2 8191953

[B351] Yanagihara R. Ajdukiewicz A. B. Garruto R. M. Sharlow E. R. Wu X. Y. Alemaena O. (1991). Human T-lymphotropic virus type I infection in the Solomon Islands. Am. J. Trop. Med. Hyg. 44, 122–130 2012254 10.4269/ajtmh.1991.44.122

[B352] Yanagihara R. Jenkins C. L. Alexander S. S. Mora C. A. Garruto R. M. (1990). Human T lymphotropic virus type I infection in Papua New Guinea: high prevalence among the Hagahai confirmed by western analysis. J. Infect. Dis. 162, 649–654 10.1093/infdis/162.3.649 2387991

[B353] Ydy R. R. Ferreira D. Souto F. J. Fontes C. J. (2009). Prevalence of human T-cell lymphotropic virus (HTLV-1/2) infection among puerperae in Cuiaba, Mato Grosso, 2006. Rev. Soc. Bras. Med. Trop. 42, 28–32 10.1590/S0037-86822009000100007 19287932

[B354] Yim M. Hayashi T. Yanagihara E. Kadin M. Nakamura J. (1986). HTLV-I-associated T-cell leukemia in Hawaii. Am. J. Med. Sci. 292, 325–327 10.1097/00000441-198611000-00015 2877581

[B355] Zaaijer H. L. Cuypers H. T. Dudok de Wit C. Lelie P. N. (1994). Results of 1-year screening of donors in The Netherlands for human T-lymphotropic virus (HTLV) type I: significance of Western blot patterns for confirmation of HTLV infection. Transfusion 34, 877–880 10.1046/j.1537-2995.1994.34294143953.x 7940659

[B356] Zabaleta M. Peralta J. Birges J. Bianco N. Echeverria de Perez G. (1994). HTLV-I-associated myelopathy in Venezuela. J. Acquir. Immune Defic. Syndr. 7, 1289–1290 10.1097/00126334-199412000-00013 7965642

[B357] Zaninovic V. Arango C. Biojo R. Mora C. Rodgers-Johnson P. Concha M. (1988). Tropical spastic paraparesis in Colombia. Ann. Neurol. 23(Suppl.), S127–S132 10.1002/ana.410230730 2894802

[B358] Zaninovic V. Sanzon F. Lopez F. Velandia G. Blank A. Blank M. (1994). Geographic independence of HTLV-I and HTLV-II foci in the Andes highland, the Atlantic coast, and the Orinoco of Colombia. AIDS Res. Hum. Retroviruses 10, 97–101 10.1089/aid.1994.10.97 8179968

[B359] Zhuo J. Yang T. Zeng Y. Lu L. (1995). Epidemiology of anti-human T-cell leukemia virus type I antibody and characteristics of adult T-cell leukemia in China. Chin. Med. J. 108, 902–906 8728941

[B360] Zoulek G. Schatzl H. Kawabata M. de Cabral M. B. Cabello A. Freutsmiedl K. (1992). A seroepidemiological survey of antibodies to HTLV-I/HTLV-II in selected population groups in Paraguay. Scand. J. Infect. Dis. 24, 397–398 10.3109/00365549209061351 1354886

[B361] Zurita S. Costa C. Watts D. Indacochea S. Campos P. Sanchez J. (1997). Prevalence of human retroviral infection in Quillabamba and Cuzco, Peru: a new endemic area for human T cell lymphotropic virus type 1. Am. J. Trop. Med. Hyg. 56, 561–565 9180608 10.4269/ajtmh.1997.56.561

